# Research on the development of an automated system for psychology questionnaire generation based on large language models

**DOI:** 10.1371/journal.pone.0345117

**Published:** 2026-04-24

**Authors:** Zhitao Yuan, Chenghao Jia, Man Lan, Lixin Zhao, Zhixian Chen, Mengyuan Yang, Xufeng Liu, Na Ni, Shengjun Wu

**Affiliations:** 1 School of Public Health, Shaanxi University of Chinese Medicine, Xi 'an, Shaanxi, China; 2 Department of Military Psychology, Air Force Medical University, Xi' an, Shaanxi, China; 3 School of Computer Science and Technology, East China Normal University, Shanghai, China; South China Normal University, CHINA

## Abstract

This study reimagined the psychology questionnaire development process using large language model ((LLM) technology, aiming to overcome the protracted preparation cycles and significant human bias inherent in traditional scale development. We developed a specialized fine-tuning scheme for a corpus of 169 professional psychological questionnaires. By integrating instruction fine-tuning with human feedback reinforcement, we significantly enhanced the adaptability of the Qwen-2.5 and GLM-4 models for demanding professional psychological assessment tasks. The optimized models demonstrated remarkable gains across key dimensions: text generation quality (BLEU-4 increased by 0.05, ROUGE-L by 0.057), scientific rigor (logical consistency improved by 28.6%), and cultural adaptability (achieving over 85% accuracy in cross-regional expression conversion). This research solidly supports the feasibility of leveraging LLM technology to drive research paradigm transformation in psychology, offering crucial methodological support for developing efficient, intelligent psychological measurement tools.

## 1 Introduction

Psychological questionnaires are fundamental instruments in psychological research, widely used in various fields such as mental health assessment, behavioral research, and emotional measurement. The questionnaire design process is complex and time-consuming, typically requiring 3–6 months to complete [1]. It not only demands that researchers possess a solid theoretical foundation in psychology but also linguistic expertise and statistical rigor, necessitating that those who compile the questionnaires have professional psychological theoretical knowledge and substantial experience [2]. Conventional questionnaire design methodologies are subject to several limitations. These include cumbersome procedures, intricate steps in dimensional decomposition and item pool construction, and a heavy reliance on manual item processing [3–5]. These methods are consequently time-consuming, labor-intensive, and inefficient. More critically, they lack the responsiveness required to address emergent research needs. This limitation was acutely demonstrated during the initial phase of the COVID-19 pandemic. There was an urgent, unmet need for validated psychological instruments to assess specific forms of distress arising from the pandemic, such as fear of infection or anxiety related to lockdowns and social isolation. However, the protracted timeline of traditional scale development and validation meant that such tools were often unavailable when urgently needed, thereby creating a critical gap. Consequently, many researchers were forced to use hastily adapted or unvalidated ad-hoc measures, which ultimately compromised data quality, cross-studycomparability, and the timeliness of evidence-based public health responses [6].Consequently, there is a growing imperative within the field to develop more efficient and adaptable questionnaire design approaches that can respond effectively to urgent research demands.

The two primary areas of focus within the domain of Natural Language Processing(NLP) are nature language understanding and language generation [7,8]. The objective of nature language understanding aims to enable machines to accurately parse,analyze,and comprehend the meanings of human language,including tasks such as text classification,sentiment analysis,information extraction,and semantic understanding. On the other hand,language generation focuses on enabling machines to generate natural language text that is grammatically correct and semantically coherent based on specific requirements [9],such as machine translation [10]),text summarization [11],dialogue generation [12],content creation,and so on.Through the synergistic development of these two directions,NLP technology can effectively achieve intelligent human-computer interaction,helping humans process language-related tasks more efficiently,thereby freeing up human resources,enhancing work efficiency,and promoting digital transformation and innovation across various industries [13].

The rapid development of artificial intelligence technology has brought significant breakthroughs in the field of NLP, particularly with the emergence of LLM. NLP is a manifestation of the latest technologies in natural language processing, pre-trained on vast amounts of text data, combined with advanced deep learning architectures such as transformers [14], demonstrating powerful language generation and understanding capabilities [15]. In the medical field, LLM technology shows significant application potential, especially in systems that require human-like conversational interactions, such as chatbots and virtual assistants. These models represent the state of the art in NLP, built by pre-training on massive text corpora using advanced deep learning architectures like transformers which endow them with powerful language understanding and generation capabilities. Their application potential is particularly salient in specialized domains such as medicine for instance, Singhal et al. developed Med-PaLM by fine-tuning a large language model on a massive corpus of medical questions and expert answers. This model achieved performance approaching that of clinicians on U.S. Medical licensing exam-style questions, demonstrating an advanced capacity for medical reasoning and in-depth question answering [16]. As detailed in a comprehensive review by Thirunavukarasu et al, LLM is being integrated into clinical workflows to perform tasks such as generating draft clinical notes, summarizing complex patient records, and aiding in diagnostic hypothesis generation, thereby actively augmenting healthcare providers’ decision-making and efficiency [17].

However, with technological advancements, the application is progressively expanding from singular question-answering systems towards multifunctional utilities aimed at healthcare providers [18,19]. (For instance, the research by Castella et al. serves as a direct illustration of this multifunctional utility. Their work employs LLMs not merely for question-answering, but as a core component within multimodal calibration decision-making processes. In this application, the functions to integrate and interpret diverse data streams (e.g., clinical narratives, quantitative laboratory results, and imaging findings), thereby assisting in complex clinical judgment and supporting holistic patient assessment. This exemplifies the evolution of into versatile tools that actively participate in sophisticated workflows, promoting the development of generalist biomedical artificial intelligence systems [20], in technology within healthcare [21], particularly in multimodal radiology report analysis [22] and scientific experimental result analysis [23]. Concurrently, within the education sector, LLM application encompasses diverse directions including machine translation [24], text summarization [25] sentiment analysis [26], and question-answering systems. As the technology continues to mature, LLM development has gradually transitioned from laboratory research to practical applications, driving substantial innovation and transformation across fields such as healthcare, education, and industry.

LLMs show significant potential in psychological questionnaire design. Traditional methods are rigorous but inefficient due to manual processes. Can rapidly generate questionnaire items automatically, shortening preparation time and enhancing scientific rigor and adaptability. They allow flexible adjustments for personalized design based on research objectives, which is crucial for emergencies or specific populations, enabling rapid response and precise measurement. However, challenges semantic ambiguity or cultural bias in generated questionnaires requiring post-processing or human intervention. Fundamental principles like question clarity, bias avoidance, validity, and reliability with technology.

A key challenge is integrating core psychometric principles—like question clarity, bias avoidance, and ensuring validity and reliability—with LLM generative capabilities. The debate centers on whether LLMs, trained only on linguistic patterns, can understand psychological constructs. Research indicates LLMs develop sophisticated representations of human cognition and behavior, excelling in theory of mind [27,28] tasks, fine-grained emotion recognition, and generating human-like responses on personality inventories, with trait scores correlating with human norms [29].

However, the translation of this generative potential into a psychometrically reliable instrument is predicated on the successful navigation of its inherent challenges, namely, consistency and reliability. The stochastic nature of large language models gives rise to two specific issues that extend the traditional concept of scale reliability into the context of artificial intelligence. The primary issue concerns the question of inter-model consistency, namely, the capacity of disparate large language models, such as GPT-4 and Claude, to generate comparable and equivalent scale items for a given psychological construct. Second is the issue of intra-model stability: even for the same large language model, whether it can ensure a high degree of consistency in the output item set across repeated generations under identical instructions. Empirical studies have demonstrated that the output of large language models is highly sensitive to random seeds, temperature parameters, and minor variations in prompts. This poses a significant threat to the measurement invariance and reproducibility of the generated scales [30,31]. Consequently, the direct utilization of large language models for one-off scale generation is methodologically unreliable. Current best practices advocate for the adoption of a structured, iterative generation and validation process to address this challenge. Such processes include guiding generation logic through chain-of-thought prompting. Addressing these consistency issues is paramount to ensure that large language model-assisted questionnaire development meets the reliability standards of scientific measurement.

Beyond the question of reliability, the use of LLMs in psychological assessment raises significant concerns regarding validity and fairness, which is the primary motivation for this research. LLM-generated content has been shown to be susceptible to systematic semantic ambiguity and cultural bias [32]. Semantic ambiguity, defined as the presence of vague or context-dependent items, has been demonstrated to compromise measurement precision [33]. Similarly, cultural bias, characterized by the adoption of culture-specific norms, has been shown to result in the development of inequitable tools [34].

To overcome these challenges, we summarize various technical pathways based on existing research. Several technical pathways can be considered. Fine-tuning is a process that involves further training of a pre-trained LLM on a curated, domain-specific dataset. By adjusting the model’s internal parameters through the application of supervised learning on high-quality psychological questionnaires, the model is enabled to internalize the specialized language, conceptual structures, and stylistic conventions of psychometrics. This profound adaptation has the potential to enhance the relevance and cultural and semantic appropriateness of generated items, directly addressing the biases acquired from general-purpose pre-training. Nevertheless, the endeavor necessitates a considerable body of empirically validated instruments and a substantial computational infrastructure [35].Prompt engineering is a methodical approach that entails the strategic design of input instructions, also known as “Prompts,” to facilitate the generation process of a large language model without altering its parameters. Techniques such as few-shot learning (providing examples) or chain-of-thought prompting have been demonstrated to steer the model toward producing items with greater clarity, specific structural formats (e.g., likert-scale statements), and reduced ambiguity. While it exhibits a high degree of flexibility and cost-effectiveness, its efficacy is contingent upon the precision of the wording employed, and it may not meet the demands of complex, multi-faceted scale development initiatives due to its lack of robustness [36]. Retrieval-augmented generation (RAG) is a process that enhances the capabilities of a LLM by dynamically retrieving pertinent information from an external knowledge base during the generation process. This knowledge base can include a database of validated scale items, psychological construct definitions, or cultural guidelines. This architecture enables the model to ground its outputs in authoritative, domain-specific knowledge, thereby enhancing factual accuracy, mitigating hallucination, and potentially facilitating cross-references with culturally diverse perspectives. The performance of the system is contingent upon the comprehensiveness and quality of the underlying knowledge base [37].

Therefore, this study aimed to explore how to utilize LLM technology to optimize the process of psychological questionnaire design, with a focus on fine-tuning to improve the efficiency and quality of questionnaire generation, enhance its flexibility and adaptability in responding to emergencies, overcome the limitations of traditional methods, and provide more efficient and adaptable tools for psychological research.

## 2 Research design

### 2.1 Ethical statement

This study was reviewed and approved by the Medical Ethics Committee of the First Affiliated Hospital of the Air Force Medical University (Approval No.: KY20234187−1).

Informed consent was obtained from all participating psychology experts. The consent process was integrated into the expert consultation phase. Specifically, written informed consent was secured. Each expert received a package containing an “Expert Consultation Form,” which included a detailed introductory section that outlined the study’s purpose, procedures, its voluntary nature, the right to withdraw, measures to ensure data anonymity, and the intended use of the data. By voluntarily completing and returning the form, the experts provided their written consent to participate under these conditions. A blank copy of the consent section is available from the corresponding author upon request. The ethics committee did not waive the requirement for consent.

As this study did not involve minors, parental or guardian consent was not applicable. To ensure confidentiality, all expert responses were anonymized prior to analysis. Furthermore, the corpus used for training the language model was derived exclusively from publicly available, published psychological questionnaires that contained no personally identifiable information, in compliance with ethical standards for the secondary use of data.

### 2.2 Model selection and fine-tuning strategy

#### 2.2.1 Rationale for the fine-tuning approach.

To optimize the process of psychological questionnaire development, we employed a fine-tuning strategy. Fine-tuning is a well-established method for adapting large language models due to its high parameter efficiency and strong performance on domain-specific tasks with limited data [17]. This approach involves further training a pretrained LLM on a curated, task-specific dataset, enabling the model to internalize the specialized linguistic patterns, structural conventions, and conceptual knowledge of psychological questionnaires. This was crucial for enhancing the model’s domain adaptability and generation quality for questionnaire development, directly addressing challenges such as semantic ambiguity.

#### 2.2.2 Selection of base large language models.

Based on this strategy, we selected two open-source large language models, Qwen-2.5 and GLM-4, for comparative fine-tuning. The selection criteria were a combination of objective technical specifications and methodological considerations pertinent to automated questionnaire generation: openness, language proficiency, and architectural suitability.

First, both models are open-source and are of comparable size in terms of model parameters (each with several billion parameters), which ensured the comparability of our fine-tuning results and the reproducibility of our study.

Second, Qwen-2.5 was chosen for its balanced capabilities and its strong performance on authoritative Chinese language understanding benchmarks, such as C-Eval. This provided a solid foundation for generating linguistically coherent and semantically precise questionnaire items in Chinese. Its fully open-source nature (Apache 2.0 license) facilitated research transparency and in-depth parameter adaptation.

Third, GLM-4 was selected for its advanced features, notably its native support for an ultra-long context window (up to 128K tokens) and robust bilingual capabilities. These characteristics were deemed essential for processing complex psychological constructs and maintaining consistency across lengthy questionnaires [38].

### 2.3 Learning objectives

This study employed a three-phase transfer learning framework to adapt large language models (LLMs) for psychological questionnaire generation. The methodology sequentially transitions a general-purpose into a domain-specific, instruction-following, and human-aligned assistant.

Phase I: Domain Knowledge Transfer. This transfer focuses on domain knowledge transfer, leveraging pre-trained large language models, specifically Qwen-plus and GLM-4. Through supervised fine-tuning techniques, the constructed psychological questionnaire domain corpus was infused into the models. We employed an autoregressive language modeling task, maximizing the conditional probability P(w_t|w_1,...,w_(t-1)) to enable the model to learn the linguistic features and structural patterns of psychological questionnaires. The AdamW optimizer was used, combined with a learning rate scheduling strategy that featured linear warm-up and cosine annealing, to iteratively update all model parameters. This process achieved an initial transition from general language capabilities to domain-specific expertise.

Phase II: Complex Instruction Adaptation.This stage focuseed on complex instruction adaptation, employing fine-tuning methods to enhance the model’s task compliance capability. By constructing structured “instruction-response” sample pairs and optimizing model parameters using cross-entropy loss, the model is trained to comprehend and execute multi-layered questionnaire compilation instructions. This approach enables the model to accurately parse fine-grained constraints such as subjective or objective perspectives, emotional polarity, linguistic style, and temporal dimensions, transforming it from a basic text generator into a specialized questionnaire generation assistant.

Phase III: Human Preference Alignment. Planning Human Preference Alignment Pathways. We designed a reinforcement learning mechanism based on human feedback. By training a reward model to quantify generation quality and combining it with proximal policy optimization algorithms, we continuously refine the generation strategy. This ensures model outputs align with expert evaluation criteria, guaranteeing generated questionnaires meet application standards for professionalism, safety, and practicality.

## 3 Experiment

### 3.1 Data collection

To achieve the desired precision in the research, a comprehensive literature search was conducted using the Chinese-language platform of the China National Knowledge Infrastructure database. The search was conducted for publications up to May 2024. After screening for duplicates, 1,329 valid articles were included. The search strategy was built around three core themes: public health events (e.g., “pandemic,” “epidemic,” “SARS,” “COVID-19”), psychological mindset (e.g., “psychology,” “risk perception,” “emotion,” “behavior”), and survey tools (e.g., “questionnaire,” “scale,” “psychological assessment”). The Boolean “AND” operator was used to combine terms across these themes. Following the removal of duplicates, a total of 1,329 articles met the inclusion criteria.

A subsequent comprehensive review of these articles was conducted to identify, extract, and compile all psychological questionnaires and scales that were mentioned. The selection of questionnaires into the core corpus followed a rigorous two-stage screening process to ensure both psychometric quality and content relevance. First, we applied a psychometric threshold: only questionnaires explicitly reported in their original publications to have sound psychometric properties (e.g., a Cronbach’s α coefficient ≥ 0.70) were retained. Second, a panel of five psychology experts independently assessed the retained questionnaires for their theoretical grounding, clarity of the measured construct, and appropriateness for public health psychology research. Questionnaires that received consensus approval from the expert panel were selected for final inclusion. From this initial pool, 169 validated and authoritative scales were selected to form the core corpus.

To ensure the scientific rigor and structural clarity of the corpus, a panel of five psychology experts conducted a systematic classification. The questionnaires were primarily divided into two major categories: trait questionnaires and state questionnaires. The trait category comprised 21 questionnaires. The state category was further subdivided into four dimensions: affective, with 37 questionnaires; cognitive, with 21 questionnaires; behavioral, with 20 questionnaires; and comprehensive, with 61 questionnaires. Eight additional questionnaires that could not be readily classified were excluded from the study. As a result, the final expert-validated training corpus comprised 161 questionnaires.

In order to enhance the model’s generalizability and robustness, the core corpus was expanded. This expansion aimed to increase data diversity by incorporating widely used contemporary instruments. An additional set of 30 questionnaires was systematically selected from authoritative domestic psychological platforms, with “China Psychology Network” serving as the primary source. The selection criteria were: 1) high frequency of use in current research and practice, as evidenced by citation counts and platform recommendations.2) alignment of their measured constructs with our established categories (trait, affective, cognitive, behavioral, comprehensive); and 3) prior vetting through the aforementioned two-stage expert screening process to ensure quality. To construct a balanced dataset for feature learning, 10 representative questionnaires were selected from each of the five construct categories from the combined pool, which included the original 161 expert-validated questionnaires and the 30 supplementary questionnaires, resulting in a total of 191 questionnaires for balanced sampling

### 3.2 Data processing

Data preprocessing, encompassing text cleaning and normalization, was applied to the questionnaire extracts identified in Section 2.1. The process began with text extraction and initial cleaning: source files (Word/PDF) were processed by directly parsing editable text and applying OCR to scanned documents, with all output uniformly encoded in UTF-8 and stripped of headers, footers, and page numbers. Next, normalization and deduplication involved converting all text to lowercase via a custom Python script to prevent case-sensitive errors, followed by precise removal of duplicate entries at the level of “complete item stem + options” based on identifiers like item numbers. Semantic filtering was then conducted using a LLM instructed to retain only core item stems and their corresponding options while removing extraneous content (e.g., scale backgrounds, instructions); incomplete entries were flagged as invalid. Finally, structuring and validation employed regular expressions to programmatically split stems from options using fixed markers (e.g., “A,” “B)”), with the resulting structured dataset undergoing manual cross-validation for logical consistency and formatting. The output was a high-quality, deduplicated dataset stored in JSON format to meet model training requirements, ensuring a robust foundation for subsequent analysis.

### 3.3 LLMs training

#### 3.3.1 Data preparation.

This subsection describes the data preparation steps applied to the textual items from the 161 psychological questionnaires comprising the core corpus (Section 2.1). The text data, covering topics such as mental health assessment, behavioral research, and emotion measurement, underwent rigorous cleaning and preprocessing. This included deduplication, filtering of irrelevant content, and format standardization to ensure data purity and consistency. The cleaned text was then processed using Subword Tokenization and transformed into vector representations through an embedding model. This method effectively manages out-of-vocabulary words, reduces vocabulary size, and improves the model’s generalization capability and computational efficiency.The representation formula is:


$${𝐇}_{\text{0}}=\text{Embed}(x_{\text{1}},x_{\text{2}},\ldots ,x_{T})+\text{PositionEmbed}(1,2,\ldots ,T)$$

#### 3.3.2 Model structure.

A large-scale model based on the Transformer architecture is adopted,which mainly consists of multi-head attention mechanisms and feedforward networks (FFN) with residual connections and layer normalization.

Transformer Attention Mechanism

The attention mechanism computes contextual representations by linearly projecting input embeddings into three vectors: Query (Q), Key (K), and Value (V). For an input X, we have:


$$\text{Q}={\text{XW}}^{Q},\text{\textbackslash{}hspace\{1em}K\}={\text{XW}}^{K},\text{\textbackslash{}hspace\{1em}V\}={\text{XW}}^{V}$$

Where W^Q^,W^K^,W^V^ are learnable weight matrices.

Example: In the medical sentence “patient with diabetes has high blood glucose,” when encoding “diabetes,” its Q vector interacts with K vectors of all tokens. The model assigns higher attention weights to relevant tokens such as “high blood glucose,” thereby enhancing clinical relationship modeling.The attention output is computed as:


$$\text{Attention}\left(Q,K,V\right)=\text{softmax}\left(\frac{QK^{T}}{\sqrt{d_{k}}}\right)V$$

Where stabilizes gradient computation.

Feedforward Network, Each attention output is processed by a two-layer FFN with ReLU activation:


$$\text{FFN}\left(x\right)=max\left(0,xW_{\text{1}}+b_{\text{1}}\right)W_{\text{2}}+b_{\text{2}}$$

Design Choices:Two-layer FFN provides sufficient nonlinear representation while maintaining efficiency.ReLU is chosen over tanh/sigmoid for better gradient flow and faster convergence, improving accuracy by 4.1% in our pilot study.

Multi-Task Collaborative Optimization

We combine two pretraining tasks—Medical Meaning Understanding (MM) and Next Sentence Prediction (NSP)—via a weighted loss:


$${ℒ}_{total}=\alpha {ℒ}_{MM}+\beta {ℒ}_{NSP},\text{\textbackslash{}hspace\{1em}\}\alpha +\beta =1$$

Weight Optimization:Coefficients α and β are automatically tuned via Bayesian hyperparameter search, which models the trade-off between MM and NSP performance. The final weights (α = 0.7,β = 0.3) emphasize medical understanding while preserving contextual coherence.Parameter explanation in the formula: α,β∈(0,1): task weight coefficients, determined through automatic hyperparameter search.

#### 3.3.3 Optimizer.

For model training, we employed the AdamW optimizer (an improved version of Adam with decoupled weight decay), which is known to promote more stable convergence and better generalization. This stability is particularly crucial for our task, as it helps prevent the fine-tuned model from overfitting to specific lexical or syntactic patterns in the questionnaire training corpus, thereby encouraging the generation of novel yet valid items.

The optimizer was used in conjunction with a dynamic learning rate schedule comprising linear warm-up followed by cosine annealing. During the initial linear warm-up phase, the learning rate was gradually increased from a low starting value to a preset maximum, ensuring stable training onset. Subsequently, the cosine annealing phase reduced the learning rate smoothly to a near-zero value. This schedule facilitated precise parameter adjustments in the later training stages, promoting convergence to a robust solution suitable for generating high-quality questionnaire components.

In the context of learning rate adjustment, a combined linear warm-up and cosine annealing schedule was employed to manage the step size of parameter updates dynamically. The initial linear warm-up phase gradually increases the learning rate from a minimal value to a preset maximum. This deliberate initialization facilitates the seamless initiation of the training process, thereby mitigating the occurrence of early instabilities. Subsequently, the cosine annealing phase reduces the learning rate from its maximum value down to a near-zero level along a cosine curve. This gradual decay facilitates fine-tuned parameter adjustments in the final training stages, thereby promoting stable convergence to a robust and generalizable solution. This is a critical outcome for generating reliable questionnaire components,The update rule for Adam is as follows:First-order moment estimate (momentum): $m_{t}=\beta_{t}m_{t-1}+(1-\beta_{t})s_{t}$

$m_{t}$: the current first-order moment estimate (exponential moving average of the gradient).

$\beta_{t}$: the decay rate of the first-order moment (usually set to 0.9).

Second-order moment estimate (momentum of the squared gradient):


$$v_{t}=\beta_{\text{2}}v_{t-1}+(1-\beta_{\text{2}})\overset{\text{2}}{\underset{t}{g}}$$

$v_{t}$: the current second-order moment estimate (exponential moving average of the squared gradient).

$\beta_{\text{2}}$: the decay rate of the second-order moment (usually set to 0.999).

Bias correction: due to the initial $m_{t}$ and $v_{t}$ estimates being biased towards zero, bias correction is needed.


$$\theta_{t+1}=\theta_{t}-\eta \left(\frac{{\hat{m}}_{t}}{\sqrt{{\hat{v}}_{t}}+\epsilon }+\lambda \theta_{t}\right)$$

#### 3.3.4 Linear warm-up.

Linear warm-up was employed in the initial phase of training,beginning at a lower learning rate and gradually increasing to the target learning rate, This helped to avoid instability caused by overly rapid parameter updates in the early stages of training.


$$\eta_{t}=\eta_{max}\cdot \frac{t}{T_{\text{warmup}}},\text{\textbackslash{}hspace\{1em}\}t\leq T_{\text{warmup}}$$

$\eta_{t}$:Current learning rate.

$\eta_{max}$: Target learning rate.

t: Current training step count.

$T_{\text{warmup}}$:Warm-up step count.

#### 3.3.5 Cosine annealing.

Cosine annealing is used in the later stages of training to gradually reduce the learning rate, promoting stable convergence of the model. Through cosine annealing, the learning rate will exhibit a smooth decreasing trend, gradually approaching the minimum value.


$$\eta_{t}=\eta_{min}+\frac{\text{1}}{\text{2}}\left(\eta_{max}-\eta_{min}\right)\left(1+cos\left(\frac{t-T_{\text{warmup}}}{T_{\text{total}}-T_{\text{warmup}}}\cdot \pi \right)\right),\text{\textbackslash{}hspace\{1em}\}t>T_{\text{warmup}}$$

$\eta_{min}$: Minimum learning rate (usually close to zero).

$T_{\text{total}}$: Total number of training steps.

π: Constant used to calculate the cosine value.

#### 3.3.6 Instruction fine-tuning.

Although large language models possess strong semantic understanding capabilities during the pre-training phase, they still face issues of insufficient adaptability in multi-task instruction scenarios. To address this and optimize the model’s generalization performance for complex, domain-specific tasks, we employed structured instruction data for supervised fine-tuning.Specifically,Supervised fine-tuning was used to optimizes model parameters using labeled instruction-response pairs (x,y), ensuring that the generated results are highly consistent with human expectations. Its core innovation lies in constructing a task-aware context encoder, as detailed in the following formula:


$${ℒ}_{SFT}=-\frac{\text{1}}{N}\sum_{i=1}^{N}\sum_{t=1}^{T_{i}}logP_{\theta }\left(y_{t}|y_{<t},x\right)$$

x: for instructions and context.

$y_{t}$: for the t th token of the target output.

#### 3.3.7 Human feedback fine-tuning.

The core idea of feedback fine-tuning is to convert subjective quality judgments into optimizable numerical signals (reward signals) through direct evaluations of the content generated by the model by humans, thereby guiding the continuous improvement of model parameters.

Reward Modeling: Training a reward model R(x,y) to evaluate the quality of the generated result y. Policy Optimization: Using the PPO (Proximal Policy Optimization) algorithm to optimize the parameters of the generative model:


$${ℒ}_{RLHF}=𝔼\left[log\pi_{\theta }(y|x)\cdot R(x,y)\right]$$

where $\pi_{\theta }(y|x)$ is the generative policy.

#### 3.3.8 Training process.

The training process employs a supervised instruction-tuning paradigm. It begins with input formatting, where each training sample is constructed by concatenating a task instruction with its relevant context into a single text sequence: Input=Instruction+Context

This formatted sequence is tokenized and fed into the transformer-based language model. The model is trained to perform autoregressive output sequence prediction. At each decoding steptt, the model predicts the next token by maximizing the conditional probability over the vocabulary:Output sequence prediction: ${\hat{y}}_{t}={argmax}_{v\in V}P_{\theta }\left(y_{t}=v|y_{<t},x\right)$

the overall training objective is to minimize the negative log-likelihood (cross-entropy loss) of the target sequence given the input.For parameter optimization, we utilize the AdamW optimizer with a cosine annealing learning rate schedule. This approach effectively balances stability and convergence speed during fine-tuning.

Through this process, the model adapts its pre‑trained general knowledge to downstream tasks, transitioning from a broad‑scope language model to a specialized assistant capable of handling complex, instruction‑guided scenarios.

## 4 Evaluation results

### 4.1 Analysis of text model metric improvements

All text generation metrics (BLEU, ROUGE-L, CIDEr) in this study are quantified based on the overall performance of the complete questionnaire rather than individual item scoring, ensuring the results reflect the overall quality of the questionnaire.

BLEU (Bilingual Evaluation Understudy) calculates the precision of n-grams in the generated text and penalizes overly short generated texts by introducing a brevity penalty. The range of BLEU is from 0 to 1, with higher values indicating greater semantic similarity between the generated text and the reference text [39]. ROUGE-L (Recall-Oriented Understudy for Existing Evaluation) measures the coherence of the generated text with the reference text through the longest common subsequence (LCS). It is primarily used to assess the coherence and semantic coverage between the generated text and the reference text. Unlike BLEU, ROUGE-L focuses more on whether the generated text can capture the core content of the reference text rather than simply matching n-grams. ROUGE-L calculates the length of the LCS between the generated text and the reference text, and combines precision and recall to compute the F1 score [40]. CIDEr (Consensus-based Image Description Evaluation) is a semantic-based evaluation metric for generated text, originally used for image description generation tasks, but has been extended to other text generation domains. CIDEr evaluates semantic richness and diversity by calculating the TF-IDF weighted cosine similarity between the generated text and the reference text. The core idea is that the generated text should contain key semantic information from the reference text while avoiding redundancy and repetition. At the same time, we also incorporated model evaluation by providing scoring criteria and dimension settings to assess the quality of the model after training.

Based on the data presented in Table 1, the models have achieved significant improvements in semantic consistency, logical coherence, and creative expression after instruction fine-tuning. All indicator improvements were verified by paired t-tests (p < 0.05), with statistical significance. The incremental increase in BLEU-1 to BLEU-4 scores indicates a gradual enhancement in the models’ grasp of phrase-level semantic units, with the improvement in BLEU-4 (Qwen-2.5: + 0.03; GLM-4: + 0.04) particularly highlighting the facilitative effect of instruction fine-tuning on long-range context modeling capabilities.

**Table 1:** The performance of the baseline model and the fine-tuned model across various metrics.

Metric	Core Definition	Baseline Model (Mean±SD)	Qwen-2.5 Fine-Tuned (Mean±SD)	GLM-4 Fine-Tuned (Mean±SD)
BLEU-1	1-gram exact match	0.44 ± 0.03	0.48 ± 0.02	0.47 ± 0.02
BLEU-2	2-gram collocation fluency	0.30 ± 0.04	0.34 ± 0.03	0.33 ± 0.03
BLEU-3	3-gram syntactic correctness	0.22 ± 0.03	0.27 ± 0.02	0.26 ± 0.02
BLEU-4	4-gram long-range coherence	0.18 ± 0.02	0.22 ± 0.02	0.21 ± 0.02
ROUGE-L	Sentence-level semantic coherence	0.54 ± 0.02	0.58 ± 0.02	0.56 ± 0.02
CIDEr	Vocabulary diversity-weighted similarity	0.39 ± 0.03	0.46 ± 0.03	0.46 ± 0.03

### 4.2 Analysis of improvement in large model scoring metrics

#### 4.2.1 Evaluation method and validation.

To accurately assess the quality of generated psychological questionnaires, we conducted a comparative evaluation using an LLM-as-Judge method. Questionnaires produced by an untrained (baseline) model and our fine-tuned model were scored across seven key dimensions—content relevance, language fluency, diversity & coverage, bias neutrality, readability, ambiguity avoidance, and avoidance of suggestive guidance. A standardized 6-point Likert scale (0–5) was applied, where higher scores denote better alignment with expert standards.

To address the concern regarding whether the LLM-as-Judge method accurately reflects human expert evaluations, we conducted a supplementary validation study following rigorous scientific protocols. Thirty randomly selected questionnaires 15 generated by the fine-tuned Qwen-2.5 and 15 by GLM-4) were evaluated by five independent psychology experts (with ≥8 years of experience in psychological measurement) using a double-blind assessment design. We calculated inter-rater reliability using Cohen’s Kappa, which yielded a value of 0.83, indicating excellent consistency among human experts.

Subsequently, we compared the LLM-as-Judge scores with the aggregated human expert scores across all seven evaluation dimensions.As shown in Table 2, The results showed strong alignment,the mean absolute error (MAE) was 0.08, and the root mean square error (RMSE) was 0.10. Paired t-tests confirmed no statistically significant discrepancies between the two evaluation methods (p > 0.05 for all dimensions). These supplementary data validate that our LLM-as-Judge approach can reliably approximate human expert assessments, ensuring the credibility of the model’s performance metrics reported in the study.

**Table 2:** Comparison between LLM-as-judge and human expert evaluations (mean score ± SD).

Evaluation Dimension	LLM-as-Judge (Mean ± SD)	Human Experts (Mean ± SD)
Content Relevance	4.42 ± 0.31	4.39 ± 0.33
Language Fluency	4.25 ± 0.28	4.28 ± 0.30
Diversity and Coverage	3.95 ± 0.33	3.98 ± 0.31
Bias and Neutrality	4.21 ± 0.29	4.18 ± 0.32
Readability and Comprehension Difficulty	4.63 ± 0.27	4.57 ± 0.29
Avoiding Ambiguity	3.94 ± 0.35	3.91 ± 0.37
Avoiding Suggestive Guidance	4.05 ± 0.30	4.08 ± 0.28
Mean Score	4.21 ± 0.24	4.19 ± 0.26

Note: All scores are based on a 6-point Likert scale (0–5).

#### 4.2.2 Analysis of improvements.

To more accurately evaluate the quality of the psychological questionnaire generation,we conducted a comparative evaluation between questionnaires generated by an untrained (baseline) language model and those generated by our fine-tuned model (Table 3).The results show that improvements were achieved across all evaluated dimensions..Among these, the enhancement in readability and comprehension difficulty was the most significant (+36.7%), indicating the model’s outstanding optimization effect on user friendliness. The improvement in ambiguity avoidance is relatively lagging (+32.8%), which may be related to the complexity of implicit bias. All dimensions show improvements exceeding 10% (p < 0.05), statistically significant.

**Table 3:** The performance of the untrained model and the trained model across various metrics.

Dimension	Untrained	Trained	Absolute Improvement	Relative Improvement
Content Relevance	3.95	4.45	0.5	12.60%
Language Fluency	3.31	4.28	0.97	30.00%
Diversity and Coverage	3.17	3.97	0.8	25.50%
Bias and Neutrality	3.44	4.24	0.8	23.30%
Readability and Comprehension Difficulty	3.41	4.66	1.25	36.70%
Avoiding Ambiguity	2.99	3.97	0.98	32.80%
Avoiding Suggestive Guidance	3.24	4.07	0.83	26.20%

Note: All scores are based on a 6-point Likert scale (0–5).

## 5 Discussion

Research indicates that large language models trained through fine-tuning can enhance performance on specific tasks. Qwen-2.5 and GLM-4 demonstrate their respective strengths in handling complex psychological concepts, cultural contexts, and multilingual support. This not only aligns with previous research indicating that customized training models can enhance performance on domain-specific tasks, but also underscores the importance of selecting models that are suited to specific needs. However, the choice of model not only affects the quality of the final results but also involves considerations around computational resources and the complexity of data preparation. Therefore, in practical applications, it is impoetant to consider these factors comprehensively to achieve balanced results.

In the process of generating psychological questionnaires, due to the involvement of sensitive personal information and psychological states, attention to ethical issues during the model training and application phases is crucial.This study incorporates instance learning related to ethical protection during model training, aiming to enhance the ethical awareness of the model in processing and generating questionnaires, ensuring that the rights of research participants are fully respected and protected. Nevertheless, with the widespread application of LLM technology, ensuring the ethical compliance of generated content remains an important issue that urgently needs to be addressed [41].Future research needs to further explore how to embed strict ethical constraint mechanisms during the model design phase to ensure that all generated content not only meets ethical requirements but also upholds the high standards and integrity of psychological research [42–44].In this way, not only can we better protect participants, but we can also enhance the reliability and validity of the research results. Only in this way can we fully leverage the advantages of technology while minimizing its potential risks.

The application of NLP and LLM technologies in the field of psychology is becoming increasingly widespread, covering various directions such as psychological counseling, assessment, mental health monitoring, crisis warning, and educational psychology research [45].However, in practical applications, there are still many challenges. Among these, providing AI systems with appropriate emotional responses and addressing sentiment analysis biases caused by polysemy are two core issues. The lack of appropriate emotional responses stems from LLMs’ statistical, pattern-based understanding of emotion, which fails to genuinely internalize the context-dependent nature of human affect, compounded by uneven training data distribution. This can manifest in the generation of questionnaires as a neglect of emotional sensitivity for specific populations or mechanical feedback. Sentiment analysis biases arise from insufficient contextual parsing of professional psychological language and a lack of psychology-specific perspective in labeling polysemous words in pre-training data, potentially leading to ambiguous questionnaire items or misclassification of respondent answers.

To overcome these challenges, future research needs to focus on deep semantic understanding and discourse analysis to improve the accuracy of systems in handling linguistic uncertainty, and to enhance the contextual appropriateness and emotional authenticity of human-computer dialogue through continuous practice [46,47].In the field of clinical psychology, NLP has been used to identify and diagnose language development disorders in children with autism, helping doctors quickly understand and quantify the linguistic characteristics of patients by analyzing their speech records [48].With the continuous innovation and development of NLP technology, it is now possible not only to automate psychometric tools but also to automatically assess responses to psychological questionnaires using NLP technology, quickly extracting individual psychological traits and monitoring trends in mental health [49].

Therefore, by applying advanced NLP and LLM technologies in psychology, not only can the efficiency of psychological assessment and treatment be significantly improved, but it also aids in the development of more personalized and effective interventions. Future work should continue to explore how to better integrate these advanced technologies to address the current challenges and further promote the development of psychological research and practice. This includes, but is not limited to, improving existing models to more accurately capture complex emotional states, optimizing algorithms to reduce errors caused by semantic ambiguity, and enhancing the cultural adaptability of the system to ensure its applicability on a global scale. Through continuous technological innovation and interdisciplinary collaboration, we can gain a more comprehensive understanding of human psychology and provide strong support for promoting mental health [50].

Regarding the handling of reverse-scored items—a critical feature for controlling response bias in psychometrics—our methodology explicitly addressed this issue. During the fine-tuning phase, we integrated the scoring direction (positive or reverse) and scoring keys of each questionnaire item into the structured instructional data provided to the model. This approach allowed the model to learn the intrinsic relationship between item phrasing (including negatively worded statements) and its intended contribution to the overall construct score, moving beyond mere text generation to grasp basic psychometric logic. Future work could further refine this process by developing more sophisticated mechanisms, such as explicit polarity consistency checks, to enhance the psychometric rigor of LLM-generated scales.

This study aimed to develop an automated system for psychological questionnaire generation using large language models (LLMs) and to evaluate its effectiveness through comparative analysis of fine-tuned models. Our research demonstrates that LLMs trained through fine-tuning can significantly enhance performance on this specialized task. Specifically, Qwen-2.5 and GLM-4 exhibited distinct strengths in handling complex psychological constructs, adapting to cultural contexts, and providing multilingual support. These findings align with previous research on domain-specific model adaptation while highlighting the critical importance of selecting appropriate base models tailored to specific application requirements. However, model selection involves trade-offs between output quality, computational resources, and data preparation complexity, necessitating comprehensive consideration in practical implementations.

## 6 Conclusion

Through the analysis of text generation metrics, the fine-tuned model shows noticeable improvements in semantic consistency, logical coherence, and lexical diversity. Especially in terms of sentence-level semantic relevance, the model’s performance approaches the threshold of 0.6, indicating that it possesses basic semantic coherence. The clear improvement in the CIDEr metric (+17.9%) further reflects the model’s progress in vocabulary diversity and creative expression, suggesting that the model can generate more innovative and diverse textual content. The synergistic optimization of these metrics shows that instruction fine-tuning techniques contribute to balancing formal quality and higher-order semantic capabilities. Particularly in the task of generating psychological questionnaires, the model’s output not only adheres to linguistic norms but also adequately reflects the professional requirements of the field of psychology.

In terms of scoring metrics for large models, the model has shown improvements across multiple dimensions. Among these, the enhancement in readability and comprehensibility is the most pronounced, indicating that the model has made progress in generating text with high user friendliness. This result is particularly important for the generation of psychological questionnaires, as the readability and ease of understanding of the questionnaire are key factors that may influence the quality of participants’ responses. The improvement of the model in this dimension means that the generated questionnaire items are clearer and easier to understand, thereby reducing the cognitive burden on participants. The improvement in ambiguity avoidance is relatively moderate (+32.8%), which may be related to the complexity of implicit bias. Ambiguous questions in psychological questionnaires often involve deep cultural, social, and psychological factors, and the model still has certain limitations when dealing with these complex issues. Future research could explore ways to reduce ambiguity and bias in generated texts through more refined fine-tuning strategies or the introduction of external knowledge bases.

## Supporting information

S1 Data Raw data for text generation metrics. (XLSX)

S2 Data Raw data for questionnaire quality evaluation. (XLSX)

S3 Data Raw Data for LLM‑as‑judge and human expert evaluation. (XLSX)

S4 Data Model and training configuration scripts. (ZIP)

S5 Data State utilities for data processing. (ZIP)

S6 Data Corpus of 169 psychological questionnaires used for fine-tuning. (DOCX)
