# Supplementary material for: Research on the development of an automated system for psychology questionnaire generation based on large language models
Source: PLoS One. 2026 Apr 24;21(4):e0345117. doi: 10.1371/journal.pone.0345117 (PMC13108753; doi:10.1371/journal.pone.0345117)
Supplement: S4 Data — (ZIP) [file pone.0345117.s004.zip › S5_Code (Model & Training Configuration)/bench_qwen.docx]

# Copyright 2025 the LlamaFactory team.

#

# Licensed under the Apache License, Version 2.0 (the "License");

# you may not use this file except in compliance with the License.

# You may obtain a copy of the License at

#

# http://www.apache.org/licenses/LICENSE-2.0

#

# Unless required by applicable law or agreed to in writing, software

# distributed under the License is distributed on an "AS IS" BASIS,

# WITHOUT WARRANTIES OR CONDITIONS OF ANY KIND, either express or implied.

# See the License for the specific language governing permissions and

# limitations under the License.

import os

from dataclasses import dataclass

from typing import Any

import fire

import torch

from peft import PeftModel

from torch.utils.data import Dataset

from transformers import DataCollatorForSeq2Seq, Qwen2_5_VLProcessor

from llamafactory.extras.constants import IGNORE_INDEX

from llamafactory.hparams import get_train_args

from llamafactory.model import load_model, load_tokenizer

from llamafactory.train.callbacks import LogCallback

from llamafactory.train.sft.trainer import CustomSeq2SeqTrainer

class DummyDataset(Dataset):

def __init__(self, size: int = 1000, seq_length: int = 1024, processor: Qwen2_5_VLProcessor = None):

self.size = size

self.seq_length = seq_length

self.vocab_size = 32768

self.processor = processor

image_token_num = 18 * 18 // (2 * 2)

image_t = 2

self.text_seqlen = seq_length // 4 # 25% text

video_seq_length = self.seq_length - self.text_seqlen - image_t * image_token_num

video_t = video_seq_length // image_token_num

self.image_size = [18 * 18 * image_t, 1176]

self.image_grid_thw = torch.tensor([[1, 18, 18]] * image_t, dtype=torch.long)

self.image_seqlen = image_t * image_token_num

self.video_size = [18 * 18 * video_t, 1176]

self.video_grid_thw = torch.tensor([[video_t, 18, 18]], dtype=torch.long)

self.video_seqlen = video_t * image_token_num

def __len__(self):

return self.size

def __getitem__(self, index: int):

input_ids = torch.randint(low=0, high=self.vocab_size, size=(self.seq_length,))

input_ids[: self.image_seqlen] = self.processor.image_token_id

input_ids[self.image_seqlen : self.image_seqlen + self.video_seqlen] = self.processor.video_token_id

attention_mask = torch.ones((self.seq_length,), dtype=torch.long)

labels = input_ids.clone()

labels[: self.image_seqlen + self.video_seqlen] = IGNORE_INDEX

pixel_values = torch.rand(self.image_size, dtype=torch.float32)

pixel_values_videos = torch.rand(self.video_size, dtype=torch.float32)

return {

"input_ids": input_ids,

"attention_mask": attention_mask,

"labels": labels,

"pixel_values": pixel_values,

"pixel_values_videos": pixel_values_videos,

"image_grid_thw": self.image_grid_thw,

"video_grid_thw": self.video_grid_thw,

}

@dataclass

class MultiModalDataCollatorForSeq2Seq(DataCollatorForSeq2Seq):

def __post_init__(self):

if isinstance(self.model, PeftModel):

self.model = self.model.base_model.model

if self.model is not None and hasattr(self.model, "get_rope_index"): # for qwen2vl mrope

self.get_rope_func = self.model.get_rope_index # transformers < 4.52.0 or qwen2.5 omni

elif self.model is not None and hasattr(self.model, "model") and hasattr(self.model.model, "get_rope_index"):

self.get_rope_func = self.model.model.get_rope_index # transformers >= 4.52.0

else:

self.get_rope_func = None

def __call__(self, features: list[dict[str, Any]]) -> dict[str, "torch.Tensor"]:

batch_pixel_values = [feature.pop("pixel_values") for feature in features]

batch_pixel_values_videos = [feature.pop("pixel_values_videos") for feature in features]

batch_image_grid_thw = [feature.pop("image_grid_thw") for feature in features]

batch_video_grid_thw = [feature.pop("video_grid_thw") for feature in features]

batch: dict[str, torch.Tensor] = super().__call__(features)

batch["pixel_values"] = torch.cat(batch_pixel_values, dim=0)

batch["pixel_values_videos"] = torch.cat(batch_pixel_values_videos, dim=0)

batch["image_grid_thw"] = torch.cat(batch_image_grid_thw, dim=0)

batch["video_grid_thw"] = torch.cat(batch_video_grid_thw, dim=0)

if self.get_rope_func is not None:

rope_index_kwargs = {

"input_ids": batch["input_ids"],

"image_grid_thw": batch["image_grid_thw"],

"video_grid_thw": batch["video_grid_thw"],

"attention_mask": (batch["attention_mask"] >= 1).float(),

}

batch["position_ids"], batch["rope_deltas"] = self.get_rope_func(**rope_index_kwargs)

if "position_ids" not in batch or batch["position_ids"].dim() != 3:

raise ValueError("Qwen2VL requires 3D position ids for mrope.")

return batch

def bench_qwen(

model_name_or_path: str = "Qwen/Qwen2-VL-7B-Instruct",

batch_size: int = 1,

seq_length: int = 2048,

liger_kernel: bool = False,

deepspeed_stage: int = 3,

):

os.environ["LLAMABOARD_ENABLED"] = "true"

os.environ["LLAMABOARD_WORKDIR"] = "output/dummy_dir"

args = {

"model_name_or_path": model_name_or_path,

"enable_liger_kernel": liger_kernel,

"stage": "sft",

"do_train": True,

"finetuning_type": "full",

"dataset": "alpaca_en_demo",

"template": "qwen2_vl",

"cutoff_len": seq_length,

"output_dir": "output/dummy_dir",

"logging_steps": 10,

"save_strategy": "no",

"save_only_model": True,

"overwrite_output_dir": True,

"per_device_train_batch_size": batch_size,

"max_steps": 1000,

"bf16": True,

"include_num_input_tokens_seen": True,

"report_to": "none",

}

if deepspeed_stage in [2, 3]:

args["deepspeed"] = f"examples/deepspeed/ds_z{deepspeed_stage}_config.json"

model_args, _, training_args, finetuning_args, _ = get_train_args(args)

tokenizer_module = load_tokenizer(model_args)

tokenizer = tokenizer_module["tokenizer"]

trainset = DummyDataset(size=100000, seq_length=seq_length, processor=tokenizer_module["processor"])

model = load_model(tokenizer, model_args, finetuning_args, training_args.do_train)

data_collator = MultiModalDataCollatorForSeq2Seq(

tokenizer=tokenizer, model=model, pad_to_multiple_of=8, label_pad_token_id=IGNORE_INDEX

)

trainer = CustomSeq2SeqTrainer(

model=model,

args=training_args,

finetuning_args=finetuning_args,

data_collator=data_collator,

callbacks=[LogCallback()],

train_dataset=trainset,

**tokenizer_module,

)

trainer.train(resume_from_checkpoint=training_args.resume_from_checkpoint)

if __name__ == "__main__":

fire.Fire(bench_qwen)
