# Supplementary material for: Research on the development of an automated system for psychology questionnaire generation based on large language models
Source: PLoS One. 2026 Apr 24;21(4):e0345117. doi: 10.1371/journal.pone.0345117 (PMC13108753; doi:10.1371/journal.pone.0345117)
Supplement: S4 Data — (ZIP) [file pone.0345117.s004.zip › S5_Code (Model & Training Configuration)/eval_bleu_rouge.docx]

# Copyright 2025 the LlamaFactory team.

#

# Licensed under the Apache License, Version 2.0 (the "License");

# you may not use this file except in compliance with the License.

# You may obtain a copy of the License at

#

# http://www.apache.org/licenses/LICENSE-2.0

#

# Unless required by applicable law or agreed to in writing, software

# distributed under the License is distributed on an "AS IS" BASIS,

# WITHOUT WARRANTIES OR CONDITIONS OF ANY KIND, either express or implied.

# See the License for the specific language governing permissions and

# limitations under the License.

import json

import logging

import time

import fire

from datasets import load_dataset

try:

import jieba # type: ignore

from nltk.translate.bleu_score import SmoothingFunction, sentence_bleu # type: ignore

from rouge_chinese import Rouge # type: ignore

jieba.setLogLevel(logging.CRITICAL)

jieba.initialize()

except ImportError:

print("Please install llamafactory with `pip install -e .[metrics]`.")

raise

def compute_metrics(sample):

hypothesis = list(jieba.cut(sample["predict"]))

reference = list(jieba.cut(sample["label"]))

bleu_score = sentence_bleu(

[list(sample["label"])],

list(sample["predict"]),

smoothing_function=SmoothingFunction().method3,

)

if len(" ".join(hypothesis).split()) == 0 or len(" ".join(reference).split()) == 0:

result = {"rouge-1": {"f": 0.0}, "rouge-2": {"f": 0.0}, "rouge-l": {"f": 0.0}}

else:

rouge = Rouge()

scores = rouge.get_scores(" ".join(hypothesis), " ".join(reference))

result = scores[0]

metric_result = {}

for k, v in result.items():

metric_result[k] = round(v["f"] * 100, 4)

metric_result["bleu-4"] = round(bleu_score * 100, 4)

return metric_result

def main(filename: str):

start_time = time.time()

dataset = load_dataset("json", data_files=filename, split="train")

dataset = dataset.map(compute_metrics, num_proc=8, remove_columns=dataset.column_names)

score_dict = dataset.to_dict()

average_score = {}

for task, scores in sorted(score_dict.items(), key=lambda x: x[0]):

print(f"{task}: {sum(scores) / len(scores):.4f}")

average_score[task] = sum(scores) / len(scores)

with open("predictions_score.json", "w", encoding="utf-8") as f:

json.dump(average_score, f, indent=4)

print(f"\nDone in {time.time() - start_time:.3f}s.\nScore file saved to predictions_score.json")

if __name__ == "__main__":

fire.Fire(main)
