# Supplementary material for: Research on the development of an automated system for psychology questionnaire generation based on large language models
Source: PLoS One. 2026 Apr 24;21(4):e0345117. doi: 10.1371/journal.pone.0345117 (PMC13108753; doi:10.1371/journal.pone.0345117)
Supplement: S4 Data — (ZIP) [file pone.0345117.s004.zip › S5_Code (Model & Training Configuration)/inference.docx]

from transformers import AutoTokenizer

from vllm import LLM, SamplingParams

import sys

def inference(model_dir, prompt):

# Initialize the tokenizer

tokenizer = AutoTokenizer.from_pretrained(model_dir)

# Pass the default decoding hyperparameters of Qwen2-7B-Instruct

# max_tokens is for the maximum length for generation.

sampling_params = SamplingParams(temperature=0.7, top_p=0.8, repetition_penalty=1.05, max_tokens=512)

# Input the model name or path. Can be GPTQ or AWQ models.

llm = LLM(model=model_dir, gpu_memory_utilization=0.95, enforce_eager= True)

messages = [

{"role": "system", "content": "You are a helpful assistant."},

{"role": "user", "content": prompt}

]

text = tokenizer.apply_chat_template(

messages,

tokenize=False,

add_generation_prompt=True

)

# generate outputs

outputs = llm.generate([text], sampling_params)

# Print the outputs.

for output in outputs:

prompt = output.prompt

generated_text = output.outputs[0].text

print(f"Generated text: \n{generated_text}")

if __name__ == '__main__':

model_dir = 'model/fine_tuned_model'

prompt = sys.argv[1]

inference(model_dir, prompt)
