# Supplementary material for: Research on the development of an automated system for psychology questionnaire generation based on large language models
Source: PLoS One. 2026 Apr 24;21(4):e0345117. doi: 10.1371/journal.pone.0345117 (PMC13108753; doi:10.1371/journal.pone.0345117)
Supplement: S4 Data — (ZIP) [file pone.0345117.s004.zip › S5_Code (Model & Training Configuration)/llama_pro.docx]

# Copyright 2025 Tencent Inc. and the LlamaFactory team.

#

# This code is inspired by the Tencent's LLaMA-Pro library.

# https://github.com/TencentARC/LLaMA-Pro/blob/main/scripts/block_expansion.py

#

# Licensed under the Apache License, Version 2.0 (the "License");

# you may not use this file except in compliance with the License.

# You may obtain a copy of the License at

#

# http://www.apache.org/licenses/LICENSE-2.0

#

# Unless required by applicable law or agreed to in writing, software

# distributed under the License is distributed on an "AS IS" BASIS,

# WITHOUT WARRANTIES OR CONDITIONS OF ANY KIND, either express or implied.

# See the License for the specific language governing permissions and

# limitations under the License.

import json

import os

from collections import OrderedDict

from typing import TYPE_CHECKING

import fire

import torch

from huggingface_hub import split_torch_state_dict_into_shards

from safetensors.torch import save_file

from tqdm import tqdm

from transformers import AutoConfig, AutoModelForCausalLM, AutoTokenizer, PreTrainedModel

from transformers.modeling_utils import SAFE_WEIGHTS_INDEX_NAME, SAFE_WEIGHTS_NAME, WEIGHTS_INDEX_NAME, WEIGHTS_NAME

if TYPE_CHECKING:

from transformers import PretrainedConfig

def change_name(name: str, old_index: int, new_index: int) -> str:

return name.replace(f".{old_index:d}.", f".{new_index:d}.")

def block_expansion(

model_name_or_path: str,

output_dir: str,

num_expand: int,

shard_size: str = "5GB",

save_safetensors: bool = True,

):

r"""Perform block expansion for LLaMA, Mistral, Qwen2 or Yi models.

Usage: python llama_pro.py --model_name_or_path meta-llama/Llama-2-7b-hf --output_dir llama2_pro --num_expand 8

"""

config: PretrainedConfig = AutoConfig.from_pretrained(model_name_or_path, trust_remote_code=True)

num_layers = getattr(config, "num_hidden_layers")

if num_layers % num_expand != 0:

raise ValueError(f"`num_layers` {num_layers} should be divisible by `num_expand` {num_expand}.")

setattr(config, "num_hidden_layers", num_layers + num_expand)

config.save_pretrained(output_dir)

tokenizer = AutoTokenizer.from_pretrained(model_name_or_path, trust_remote_code=True)

tokenizer.save_pretrained(output_dir)

print(f"Expanding model of {num_layers} layers to {num_layers + num_expand} layers.")

model = AutoModelForCausalLM.from_pretrained(

model_name_or_path, torch_dtype="auto", device_map="cpu", trust_remote_code=True, low_cpu_mem_usage=True

)

assert isinstance(model, PreTrainedModel) # type hint

if save_safetensors and getattr(model.config, "tie_word_embeddings", False):

del model.lm_head # safetensors does not allow shared weights

split = num_layers // num_expand

layer_cnt = 0

state_dict = model.state_dict()

output_state_dict: dict[str, torch.Tensor] = OrderedDict()

for i in range(num_layers):

for key, value in state_dict.items():

if f".{i:d}." in key:

output_state_dict[change_name(key, i, layer_cnt)] = value

print(f"Add layer {layer_cnt} copied from layer {i}.")

layer_cnt += 1

if (i + 1) % split == 0:

for key, value in state_dict.items():

if f".{i:d}." in key:

if "down_proj" in key or "o_proj" in key:

output_state_dict[change_name(key, i, layer_cnt)] = torch.zeros_like(value)

else:

output_state_dict[change_name(key, i, layer_cnt)] = torch.clone(value)

print(f"Add layer {layer_cnt} expanded from layer {i}.")

layer_cnt += 1

for key, value in state_dict.items():

if key not in output_state_dict:

output_state_dict[key] = value

weights_name = SAFE_WEIGHTS_NAME if save_safetensors else WEIGHTS_NAME

filename_pattern = weights_name.replace(".bin", "{suffix}.bin").replace(".safetensors", "{suffix}.safetensors")

state_dict_split = split_torch_state_dict_into_shards(

output_state_dict, filename_pattern=filename_pattern, max_shard_size=shard_size

)

for shard_file, tensors in tqdm(state_dict_split.filename_to_tensors.items(), desc="Save weights"):

shard = {tensor: output_state_dict[tensor].contiguous() for tensor in tensors}

if save_safetensors:

save_file(shard, os.path.join(output_dir, shard_file), metadata={"format": "pt"})

else:

torch.save(shard, os.path.join(output_dir, shard_file))

if not state_dict_split.is_sharded:

print(f"Model weights saved in {os.path.join(output_dir, weights_name)}.")

else:

index = {

"metadata": state_dict_split.metadata,

"weight_map": state_dict_split.tensor_to_filename,

}

index_name = SAFE_WEIGHTS_INDEX_NAME if save_safetensors else WEIGHTS_INDEX_NAME

with open(os.path.join(output_dir, index_name), "w", encoding="utf-8") as f:

json.dump(index, f, indent=2, sort_keys=True)

print(f"Model weights saved in {output_dir}.")

print("- Fine-tune this model with:")

print(f"model_name_or_path: {output_dir}")

print("finetuning_type: freeze")

print(f"freeze_trainable_layers: {num_expand}")

print("use_llama_pro: true")

if __name__ == "__main__":

fire.Fire(block_expansion)
