# Supplementary material for: Research on the development of an automated system for psychology questionnaire generation based on large language models
Source: PLoS One. 2026 Apr 24;21(4):e0345117. doi: 10.1371/journal.pone.0345117 (PMC13108753; doi:10.1371/journal.pone.0345117)
Supplement: S4 Data — (ZIP) [file pone.0345117.s004.zip › S5_Code (Model & Training Configuration)/megatron_merge.docx]

# Copyright 2025 the ROLL team and the LlamaFactory team.

#

# This code is modified from the ROLL library.

# https://github.com/alibaba/ROLL/blob/main/mcore_adapter/tools/convert.py

#

# Licensed under the Apache License, Version 2.0 (the "License");

# you may not use this file except in compliance with the License.

# You may obtain a copy of the License at

#

# http://www.apache.org/licenses/LICENSE-2.0

#

# Unless required by applicable law or agreed to in writing, software

# distributed under the License is distributed on an "AS IS" BASIS,

# WITHOUT WARRANTIES OR CONDITIONS OF ANY KIND, either express or implied.

# See the License for the specific language governing permissions and

# limitations under the License.

import os

import fire

import torch

from mcore_adapter.models.converter.post_converter import convert_checkpoint_to_hf, convert_checkpoint_to_mca

from mcore_adapter.training_args import DistributingParallelArguments

from mcore_adapter.utils import get_logger

from transformers import AutoConfig

logger = get_logger(__name__)

def convert_mca_to_hf(

checkpoint_path: str,

output_path: str = "./output",

bf16: bool = False,

fp16: bool = False,

convert_model_max_length: int | None = None,

):

"""Convert megatron checkpoint to HuggingFace format.

Args:

checkpoint_path: Path to the checkpoint to convert

output_path: Path to save the converted checkpoint

bf16: Use bfloat16 precision

fp16: Use float16 precision

convert_model_max_length: Change the model_max_length in hf config.json

"""

if bf16 and fp16:

raise ValueError("bf16 and fp16 cannot be both True.")

torch_dtype = None

if bf16:

torch_dtype = torch.bfloat16

elif fp16:

torch_dtype = torch.float16

convert_checkpoint_to_hf(checkpoint_path, output_path, torch_dtype=torch_dtype)

if convert_model_max_length is not None:

config = AutoConfig.from_pretrained(output_path, trust_remote_code=True)

config.model_max_length = convert_model_max_length

config.save_pretrained(output_path)

def convert(

checkpoint_path: str,

output_path: str = "./output",

bf16: bool = False,

fp16: bool = False,

convert_model_max_length: int | None = None,

tensor_model_parallel_size: int = 1,

pipeline_model_parallel_size: int = 1,

expert_model_parallel_size: int = 1,

virtual_pipeline_model_parallel_size: int | None = None,

):

"""Convert checkpoint between MCA and HuggingFace formats.

Args:

checkpoint_path: Path to the checkpoint to convert

output_path: Path to save the converted checkpoint

bf16: Use bfloat16 precision

fp16: Use float16 precision

convert_model_max_length: Change the model_max_length in hf config.json

tensor_model_parallel_size: Tensor model parallel size

pipeline_model_parallel_size: Pipeline model parallel size

expert_model_parallel_size: Expert model parallel size

virtual_pipeline_model_parallel_size: Virtual pipeline model parallel size

"""

if bf16 and fp16:

raise ValueError("bf16 and fp16 cannot be both True.")

mca_config_path = os.path.join(checkpoint_path, "mca_config.json")

from_mca = os.path.exists(mca_config_path)

if not from_mca:

dist_args = DistributingParallelArguments(

tensor_model_parallel_size=tensor_model_parallel_size,

pipeline_model_parallel_size=pipeline_model_parallel_size,

expert_model_parallel_size=expert_model_parallel_size,

virtual_pipeline_model_parallel_size=virtual_pipeline_model_parallel_size,

)

convert_checkpoint_to_mca(

checkpoint_path,

output_path,

dist_args,

bf16=bf16,

fp16=fp16,

)

else:

convert_mca_to_hf(

checkpoint_path=checkpoint_path,

output_path=output_path,

bf16=bf16,

fp16=fp16,

convert_model_max_length=convert_model_max_length,

)

def main():

fire.Fire(convert)

if __name__ == "__main__":

main()
