# Supplementary material for: Research on the development of an automated system for psychology questionnaire generation based on large language models
Source: PLoS One. 2026 Apr 24;21(4):e0345117. doi: 10.1371/journal.pone.0345117 (PMC13108753; doi:10.1371/journal.pone.0345117)
Supplement: S4 Data — (ZIP) [file pone.0345117.s004.zip › S5_Code (Model & Training Configuration)/pissa_init.docx]

# Copyright 2025 HuggingFace Inc. and the LlamaFactory team.

#

# This code is based on the HuggingFace's PEFT library.

# https://github.com/huggingface/peft/blob/v0.11.0/examples/pissa_finetuning/preprocess.py

#

# Licensed under the Apache License, Version 2.0 (the "License");

# you may not use this file except in compliance with the License.

# You may obtain a copy of the License at

#

# http://www.apache.org/licenses/LICENSE-2.0

#

# Unless required by applicable law or agreed to in writing, software

# distributed under the License is distributed on an "AS IS" BASIS,

# WITHOUT WARRANTIES OR CONDITIONS OF ANY KIND, either express or implied.

# See the License for the specific language governing permissions and

# limitations under the License.

import os

from typing import TYPE_CHECKING

import fire

from peft import LoraConfig, TaskType, get_peft_model

from transformers import AutoModelForCausalLM, AutoTokenizer

if TYPE_CHECKING:

from transformers import PreTrainedModel

def quantize_pissa(

model_name_or_path: str,

output_dir: str,

pissa_iter: int = 16,

lora_alpha: int = None,

lora_rank: int = 16,

lora_dropout: float = 0,

lora_target: tuple = ("q_proj", "v_proj"),

save_safetensors: bool = True,

):

r"""Initialize LoRA weights with Principal Singular values and Singular vectors Adaptation (PiSSA).

Usage: python pissa_init.py --model_name_or_path path_to_model --output_dir output_dir

"""

if isinstance(lora_target, str):

lora_target = [name.strip() for name in lora_target.split(",")]

tokenizer = AutoTokenizer.from_pretrained(model_name_or_path, trust_remote_code=True)

model = AutoModelForCausalLM.from_pretrained(model_name_or_path, trust_remote_code=True, torch_dtype="auto")

lora_config = LoraConfig(

task_type=TaskType.CAUSAL_LM,

r=lora_rank,

lora_alpha=lora_alpha if lora_alpha is not None else lora_rank * 2,

lora_dropout=lora_dropout,

target_modules=lora_target,

init_lora_weights="pissa" if pissa_iter == -1 else f"pissa_niter_{pissa_iter}",

)

# Init PiSSA model

peft_model = get_peft_model(model, lora_config)

pissa_dir = os.path.join(output_dir, "pissa_init")

# Save PiSSA model

setattr(peft_model.peft_config["default"], "base_model_name_or_path", os.path.abspath(output_dir))

setattr(peft_model.peft_config["default"], "init_lora_weights", True) # don't apply pissa again

peft_model.save_pretrained(pissa_dir, safe_serialization=save_safetensors)

print(f"Adapter weights saved in {pissa_dir}")

# Save base model

base_model: PreTrainedModel = peft_model.unload()

base_model.save_pretrained(output_dir, safe_serialization=save_safetensors)

tokenizer.save_pretrained(output_dir)

print(f"Model weights saved in {output_dir}")

print("- Fine-tune this model with:")

print(f"model_name_or_path: {output_dir}")

print(f"adapter_name_or_path: {pissa_dir}")

print("finetuning_type: lora")

print("pissa_init: false")

print("pissa_convert: true")

print("- and optionally with:")

print("quantization_bit: 4")

if __name__ == "__main__":

fire.Fire(quantize_pissa)
