# Supplementary material for: Research on the development of an automated system for psychology questionnaire generation based on large language models
Source: PLoS One. 2026 Apr 24;21(4):e0345117. doi: 10.1371/journal.pone.0345117 (PMC13108753; doi:10.1371/journal.pone.0345117)
Supplement: S4 Data — (ZIP) [file pone.0345117.s004.zip › S5_Code (Model & Training Configuration)/qwen_omni_merge.docx]

# Copyright 2025 the LlamaFactory team.

#

# Licensed under the Apache License, Version 2.0 (the "License");

# you may not use this file except in compliance with the License.

# You may obtain a copy of the License at

#

# http://www.apache.org/licenses/LICENSE-2.0

#

# Unless required by applicable law or agreed to in writing, software

# distributed under the License is distributed on an "AS IS" BASIS,

# WITHOUT WARRANTIES OR CONDITIONS OF ANY KIND, either express or implied.

# See the License for the specific language governing permissions and

# limitations under the License.

"""Why we need this script for qwen_omni?

Because the qwen_omni model is constructed by two parts:

1. [Thinker]:[audio_encoder, vision_encoder, LLM backbone], which our repository does support to post-training.

2. [Talker]: [audio_decoder, wave_model], which is not supported to post-training without specific tokenizer.

When we post-training the model, we exactly train the [Thinker] part, and the [Talker] part is dropped.

So, to get the complete model, we need to merge the [Talker] part back to the [Thinker] part.

LoRA mode: [Thinker + LoRA weights] + [Original Talker] -> [Omni model]

Full mode: [Thinker] + [Original Talker] -> [Omni model]

For Processor, we do saved the processor from trained model instead of the original model.

"""

import os

import shutil

import fire

from peft import PeftModel

from transformers import AutoConfig, AutoModelForTextToWaveform, AutoProcessor

from transformers.utils import cached_file

def merge_lora(

model_path: str,

lora_path: str,

save_path: str = "./merged_model_checkpoint",

extra_file: str = "spk_dict.pt",

submodule_name: str = "thinker",

):

"""Load the original model, merge the LoRA weights.

For a specified submodule, and save the final merged model along with its configurations.

Args:

model_path (str): Path to the original model directory.

lora_path (str): Path to the directory containing LoRA weights.

save_path (str): Directory where the merged model and configurations will be saved.

extra_file (str): Name of the extra file to be copied (default: "spk_dict.pt").

submodule_name (str): Name of the submodule to merge (default: "thinker").

"""

# 1. Load the original model

model = AutoModelForTextToWaveform.from_pretrained(model_path, torch_dtype="auto", device_map="cpu")

print("Successfully loaded the original model.")

# 2. Extract the submodule to be merged (e.g., model.thinker)

if not hasattr(model, submodule_name):

raise AttributeError(f"The model does not have a submodule named '{submodule_name}'.")

base_submodule = getattr(model, submodule_name)

print(f"Successfully extracted submodule: {submodule_name}.")

# 3. Load the LoRA weights onto the extracted submodule

lora_model = PeftModel.from_pretrained(base_submodule, lora_path)

processor = AutoProcessor.from_pretrained(lora_path)

print("Successfully loaded LoRA weights and processor.")

# 4. Merge the LoRA weights into the submodule and unload the LoRA modules

merged_submodule = lora_model.merge_and_unload()

print("Successfully merged LoRA weights.")

# 5. Replace the original submodule with the merged submodule in the model

setattr(model, submodule_name, merged_submodule)

# 6. Save the final merged model along with the tokenizer and processor configuration

model.save_pretrained(save_path)

processor.save_pretrained(save_path)

print(f"Merged model and processor saved to {save_path}.")

try:

source_file = cached_file(path_or_repo_id=model_path, filename=extra_file)

shutil.copy(source_file, os.path.join(save_path, extra_file))

print(f"File '{extra_file}' copied from {model_path} to {save_path}.")

except Exception:

print(f"File '{extra_file}' not found in {model_path}, skipping copy.")

def save_full_model(

model_path: str,

thinker_path: str,

save_path: str = "./merged_model_checkpoint",

extra_file: str = "spk_dict.pt",

):

"""Load the saved thinker module and the original model, replace the thinker in the original model.

Then save the complete model along with its tokenizer and processor configuration.

Args:

model_path (str): Directory path of the original model.

thinker_path (str): Path to the saved thinker weights.

save_path (str): Directory where the merged model and configurations will be saved.

extra_file (str): Name of the extra file to be copied (default: "spk_dict.pt").

"""

# 1. Load the saved thinker module and the original model

config = AutoConfig.from_pretrained(model_path)

if getattr(config, "model_type") == "qwen2_5_omni":

from transformers.models.qwen2_5_omni import Qwen2_5OmniThinkerForConditionalGeneration # type: ignore

ThinkerClass = Qwen2_5OmniThinkerForConditionalGeneration

elif getattr(config, "model_type") == "qwen3_omni_moe":

from transformers.models.qwen3_omni_moe import Qwen3OmniMoeThinkerForConditionalGeneration # type: ignore

ThinkerClass = Qwen3OmniMoeThinkerForConditionalGeneration

else:

raise ValueError(f"Unsupported model type: {getattr(config, 'model_type')}.")

thinker = ThinkerClass.from_pretrained(thinker_path, torch_dtype="auto", device_map="cpu")

base_model = AutoModelForTextToWaveform.from_pretrained(model_path, torch_dtype="auto", device_map="cpu")

base_model.thinker = thinker

processor = AutoProcessor.from_pretrained(thinker_path)

print("Successfully loaded model weights and processor.")

# 2. Save the complete model along with its tokenizer and processor configuration

base_model.save_pretrained(save_path)

processor.save_pretrained(save_path)

print(f"Merged model and processor saved to {save_path}.")

# 3. Copy the extra file from the base model directory to the save_path

try:

source_file = cached_file(path_or_repo_id=model_path, filename=extra_file)

shutil.copy(source_file, os.path.join(save_path, extra_file))

print(f"File '{extra_file}' copied from {model_path} to {save_path}.")

except Exception:

print(f"File '{extra_file}' not found in {model_path}, skipping copy.")

if __name__ == "__main__":

fire.Fire({"save_full": save_full_model, "merge_lora": merge_lora})
