# Supplementary material for: Research on the development of an automated system for psychology questionnaire generation based on large language models
Source: PLoS One. 2026 Apr 24;21(4):e0345117. doi: 10.1371/journal.pone.0345117 (PMC13108753; doi:10.1371/journal.pone.0345117)
Supplement: S4 Data — (ZIP) [file pone.0345117.s004.zip › S5_Code (Model & Training Configuration)/train.docx]

import os

import torch

from transformers import AutoTokenizer, AutoModelForSequenceClassification, Trainer, TrainingArguments

from datasets import load_dataset, Dataset

from peft import get_peft_model, LoraConfig, TaskType

# 灏嗘枃鏈枃浠惰浆鎹负Hugging Face datasets鏍煎紡

def load_text_data(file_path):

with open(file_path, 'r', encoding='utf-8') as f:

lines = f.readlines()

return Dataset.from_dict({'text': lines})

# 鍔犺浇鏈湴妯″瀷鍜屽垎璇嶅櫒

local_model_path = '/sshfs/pretrains/Qwen/Qwen2-72B-Instruct/'

tokenizer = AutoTokenizer.from_pretrained(local_model_path)

model = AutoModelForSequenceClassification.from_pretrained(local_model_path, num_labels=2)

# 閰嶇疆LoRA

lora_config = LoraConfig(

task_type=TaskType.SEQUENCE_CLASSIFICATION,

inference_mode=False,

r=8, # rank

lora_alpha=32,

lora_dropout=0.1

)

# 搴旂敤LoRA閰嶇疆鍒版ā鍨?model = get_peft_model(model, lora_config)

# 鏁版嵁棰勫鐞嗗嚱鏁?def preprocess_data(examples):

return tokenizer(examples['text'], truncation=True, padding='max_length', max_length=128)

# 璇诲彇鏂囨湰鏁版嵁

dataset = load_text_data('path_to_your_text_file.txt')

tokenized_dataset = dataset.map(preprocess_data, batched=True)

# 璁剧疆璁粌鍙傛暟

training_args = TrainingArguments(

output_dir='./results',

num_train_epochs=3,

per_device_train_batch_size=8,

per_device_eval_batch_size=8,

warmup_steps=500,

weight_decay=0.01,

logging_dir='./logs',

logging_steps=10,

)

# 鍒涘缓Trainer瀹炰緥

trainer = Trainer(

model=model,

args=training_args,

train_dataset=tokenized_dataset,

)

# 寮€濮嬭缁?trainer.train()

# 淇濆瓨寰皟鍚庣殑妯″瀷

model.save_pretrained('model/fine_tuned_model')

tokenizer.save_pretrained('model/fine_tuned_model')
