# Supplementary material for: Research on the development of an automated system for psychology questionnaire generation based on large language models
Source: PLoS One. 2026 Apr 24;21(4):e0345117. doi: 10.1371/journal.pone.0345117 (PMC13108753; doi:10.1371/journal.pone.0345117)
Supplement: S4 Data — (ZIP) [file pone.0345117.s004.zip › S5_Code (Model & Training Configuration)/vllm_infer.docx]

# Copyright 2025 the LlamaFactory team.

#

# Licensed under the Apache License, Version 2.0 (the "License");

# you may not use this file except in compliance with the License.

# You may obtain a copy of the License at

#

# http://www.apache.org/licenses/LICENSE-2.0

#

# Unless required by applicable law or agreed to in writing, software

# distributed under the License is distributed on an "AS IS" BASIS,

# WITHOUT WARRANTIES OR CONDITIONS OF ANY KIND, either express or implied.

# See the License for the specific language governing permissions and

# limitations under the License.

import gc

import json

import time

import av

import fire

from datasets import load_dataset

from eval_bleu_rouge import compute_metrics

from tqdm import tqdm

from transformers import Seq2SeqTrainingArguments

from llamafactory.data import get_dataset, get_template_and_fix_tokenizer

from llamafactory.extras.constants import IGNORE_INDEX

from llamafactory.extras.misc import get_device_count

from llamafactory.extras.packages import is_vllm_available

from llamafactory.hparams import get_infer_args

from llamafactory.model import load_tokenizer

if is_vllm_available():

from vllm import LLM, SamplingParams

from vllm.lora.request import LoRARequest

def _need_video_kwargs(template):

NEEDED_TEMPLATE = ["qwen3_vl", "glm4v"]

if any(t in template for t in NEEDED_TEMPLATE):

return True

return False

def vllm_infer(

model_name_or_path: str,

adapter_name_or_path: str = None,

dataset: str = "alpaca_en_demo",

dataset_dir: str = "data",

template: str = "default",

cutoff_len: int = 2048,

max_samples: int | None = None,

vllm_config: str = "{}",

save_name: str = "generated_predictions.jsonl",

matrix_save_name: str = None,

temperature: float = 0.95,

top_p: float = 0.7,

top_k: int = 50,

max_new_tokens: int = 1024,

repetition_penalty: float = 1.0,

skip_special_tokens: bool = True,

default_system: str | None = None,

enable_thinking: bool = True,

seed: int | None = None,

pipeline_parallel_size: int = 1,

image_max_pixels: int = 768 * 768,

image_min_pixels: int = 32 * 32,

video_fps: float = 2.0,

video_maxlen: int = 128,

batch_size: int = 1024,

):

r"""Perform batch generation using vLLM engine, which supports tensor parallelism.

Usage: python vllm_infer.py --model_name_or_path meta-llama/Llama-2-7b-hf --template llama --dataset alpaca_en_demo

"""

if pipeline_parallel_size > get_device_count():

raise ValueError("Pipeline parallel size should be smaller than the number of gpus.")

model_args, data_args, _, generating_args = get_infer_args(

dict(

model_name_or_path=model_name_or_path,

adapter_name_or_path=adapter_name_or_path,

dataset=dataset,

dataset_dir=dataset_dir,

template=template,

cutoff_len=cutoff_len,

max_samples=max_samples,

preprocessing_num_workers=16,

default_system=default_system,

enable_thinking=enable_thinking,

vllm_config=vllm_config,

temperature=temperature,

top_p=top_p,

top_k=top_k,

max_new_tokens=max_new_tokens,

repetition_penalty=repetition_penalty,

)

)

training_args = Seq2SeqTrainingArguments(output_dir="dummy_dir")

tokenizer_module = load_tokenizer(model_args)

tokenizer = tokenizer_module["tokenizer"]

template_obj = get_template_and_fix_tokenizer(tokenizer, data_args)

template_obj.mm_plugin.expand_mm_tokens = False # for vllm generate

engine_args = {

"model": model_args.model_name_or_path,

"trust_remote_code": True,

"dtype": model_args.infer_dtype,

"max_model_len": cutoff_len + max_new_tokens,

"tensor_parallel_size": (get_device_count() // pipeline_parallel_size) or 1,

"pipeline_parallel_size": pipeline_parallel_size,

"disable_log_stats": True,

"enable_lora": model_args.adapter_name_or_path is not None,

}

if template_obj.mm_plugin.__class__.__name__ != "BasePlugin":

engine_args["limit_mm_per_prompt"] = {"image": 4, "video": 2, "audio": 2}

if isinstance(model_args.vllm_config, dict):

engine_args.update(model_args.vllm_config)

model_preparation_start_time = time.time()

llm = LLM(**engine_args)

# load datasets

dataset_module = get_dataset(template_obj, model_args, data_args, training_args, "ppo", **tokenizer_module)

train_dataset = dataset_module["train_dataset"]

sampling_params = SamplingParams(

repetition_penalty=generating_args.repetition_penalty or 1.0, # repetition_penalty must > 0

temperature=generating_args.temperature,

top_p=generating_args.top_p or 1.0, # top_p must > 0

top_k=generating_args.top_k or -1, # top_k must > 0

stop_token_ids=template_obj.get_stop_token_ids(tokenizer),

max_tokens=generating_args.max_new_tokens,

skip_special_tokens=skip_special_tokens,

seed=seed,

)

if model_args.adapter_name_or_path is not None:

lora_request = LoRARequest("default", 1, model_args.adapter_name_or_path[0])

else:

lora_request = None

# Store all results in these lists

all_prompts, all_preds, all_labels = [], [], []

need_video_kwargs = _need_video_kwargs(template)

model_predict_start_time = time.time()

# Add batch process to avoid the issue of too many files opened

for i in tqdm(range(0, len(train_dataset), batch_size), desc="Processing batched inference"):

vllm_inputs, prompts, labels = [], [], []

batch = train_dataset[i : min(i + batch_size, len(train_dataset))]

for j in range(len(batch["input_ids"])):

if batch["images"][j] is not None:

image = batch["images"][j]

multi_modal_data = {

"image": template_obj.mm_plugin._regularize_images(

image, image_max_pixels=image_max_pixels, image_min_pixels=image_min_pixels

)["images"]

}

elif batch["videos"][j] is not None:

video_metadata, video_metadata_kwargs = None, None

video = batch["videos"][j]

multi_modal_data = {

"video": template_obj.mm_plugin._regularize_videos(

video,

image_max_pixels=image_max_pixels,

image_min_pixels=image_min_pixels,

video_fps=video_fps,

video_maxlen=video_maxlen,

)["videos"]

}

if need_video_kwargs:

container = av.open(video[0], "r")

video_stream = next(stream for stream in container.streams if stream.type == "video")

sampling_indices = template_obj.mm_plugin._get_video_sample_indices(

video_stream, video_fps, video_maxlen

)

total_frames = video_stream.frames

video_metadata_kwargs = {

"fps": getattr(tokenizer_module["processor"], "video_fps", 24.0),

"do_sample_frames": False,

"total_num_frames": total_frames,

}

video_metadata = dict(

fps=video_fps,

frames_indices=sampling_indices,

total_num_frames=total_frames,

video_backend="opencv",

)

multi_modal_data["video"] = (multi_modal_data["video"], video_metadata)

elif batch["audios"][j] is not None:

audio = batch["audios"][j]

audio_data = template_obj.mm_plugin._regularize_audios(

audio,

sampling_rate=16000,

)

multi_modal_data = {"audio": zip(audio_data["audios"], audio_data["sampling_rates"])}

else:

multi_modal_data = None

vllm_input_data = {"prompt_token_ids": batch["input_ids"][j], "multi_modal_data": multi_modal_data}

if "video_metadata_kwargs" in locals() and video_metadata_kwargs is not None:

vllm_input_data["mm_processor_kwargs"] = video_metadata_kwargs

vllm_inputs.append(vllm_input_data)

prompts.append(tokenizer.decode(batch["input_ids"][j], skip_special_tokens=skip_special_tokens))

labels.append(

tokenizer.decode(

list(filter(lambda x: x != IGNORE_INDEX, batch["labels"][j])),

skip_special_tokens=skip_special_tokens,

)

)

results = llm.generate(vllm_inputs, sampling_params, lora_request=lora_request)

preds = [result.outputs[0].text for result in results]

# Accumulate results

all_prompts.extend(prompts)

all_preds.extend(preds)

all_labels.extend(labels)

gc.collect()

model_predict_end_time = time.time()

# Write all results at once outside the loop

with open(save_name, "w", encoding="utf-8") as f:

for text, pred, label in zip(all_prompts, all_preds, all_labels):

f.write(json.dumps({"prompt": text, "predict": pred, "label": label}, ensure_ascii=False) + "\n")

print("*" * 70)

print(f"{len(all_prompts)} total generated results have been saved at {save_name}.")

print("*" * 70)

# Write all matrix results when matrix_save_name is not None,

# The result matrix is referencing src.llamafactory.train.sft.workflow.run_sft # 127~132

# trainer.save_metrics("predict", predict_results.metrics)

#

# {

# "predict_bleu-4": 4.349975,

# "predict_model_preparation_time": 0.0128,

# "predict_rouge-1": 21.873359375,

# "predict_rouge-2": 4.144340625,

# "predict_rouge-l": 10.83949375,

# "predict_runtime": 131.664,

# "predict_samples_per_second": 0.076,

# "predict_steps_per_second": 0.008

# }

#

if matrix_save_name is not None:

predict_time = model_predict_end_time - model_predict_start_time

preparation_time = model_predict_start_time - model_preparation_start_time

start_time = time.time()

dataset = load_dataset("json", data_files=save_name, split="train")

dataset = dataset.map(compute_metrics, num_proc=8, remove_columns=dataset.column_names)

score_dict = dataset.to_dict()

average_score = {}

for task, scores in sorted(score_dict.items(), key=lambda x: x[0]):

score = sum(scores) / len(scores) if scores else 0.0

print(f"predict_{task}: {score:.4f}")

average_score["predict_" + task] = score

average_score["predict_model_preparation_time"] = preparation_time

average_score["predict_runtime"] = predict_time

num_steps = len(range(0, len(train_dataset), batch_size))

average_score["predict_samples_per_second"] = len(dataset) / predict_time if predict_time > 0 else 0.0

average_score["predict_steps_per_second"] = num_steps / predict_time if predict_time > 0 else 0.0

with open(matrix_save_name, "w", encoding="utf-8") as f:

json.dump(average_score, f, indent=4)

print("*" * 70)

print(f"\nDone in {time.time() - start_time:.3f}s.\nScore file saved to {matrix_save_name}.")

print("*" * 70)

if __name__ == "__main__":

fire.Fire(vllm_infer)
