# Supplementary material for: Research on the development of an automated system for psychology questionnaire generation based on large language models
Source: PLoS One. 2026 Apr 24;21(4):e0345117. doi: 10.1371/journal.pone.0345117 (PMC13108753; doi:10.1371/journal.pone.0345117)
Supplement: S5 Data — (ZIP) [file pone.0345117.s005.zip › S6_ Code (state utils)/cal_flops.docx]

# Copyright 2025 Microsoft Corporation and the LlamaFactory team.

#

# This code is inspired by the Microsoft's DeepSpeed library.

# https://www.deepspeed.ai/tutorials/flops-profiler/

#

# Licensed under the Apache License, Version 2.0 (the "License");

# you may not use this file except in compliance with the License.

# You may obtain a copy of the License at

#

# http://www.apache.org/licenses/LICENSE-2.0

#

# Unless required by applicable law or agreed to in writing, software

# distributed under the License is distributed on an "AS IS" BASIS,

# WITHOUT WARRANTIES OR CONDITIONS OF ANY KIND, either express or implied.

# See the License for the specific language governing permissions and

# limitations under the License.

import fire

import torch

from deepspeed.accelerator import get_accelerator # type: ignore

from deepspeed.profiling.flops_profiler import get_model_profile # type: ignore

from llamafactory.chat import ChatModel

def calculate_flops(

model_name_or_path: str,

batch_size: int = 1,

seq_length: int = 512,

flash_attn: str = "auto",

):

r"""Calculate the flops of pre-trained models.

Usage: python cal_flops.py --model_name_or_path path_to_model --batch_size 1 --seq_length 512

"""

with get_accelerator().device(0):

chat_model = ChatModel(dict(model_name_or_path=model_name_or_path, template="empty", flash_attn=flash_attn))

fake_input = torch.ones((batch_size, seq_length), dtype=torch.long, device=chat_model.engine.model.device)

input_dict = {"input_ids": fake_input, "labels": fake_input.clone()}

flops, macs, params = get_model_profile(

chat_model.engine.model, kwargs=input_dict, print_profile=True, detailed=True

)

print("FLOPs:", flops)

print("MACs:", macs)

print("Params:", params)

if __name__ == "__main__":

fire.Fire(calculate_flops)
