# Supplementary material for: Research on the development of an automated system for psychology questionnaire generation based on large language models
Source: PLoS One. 2026 Apr 24;21(4):e0345117. doi: 10.1371/journal.pone.0345117 (PMC13108753; doi:10.1371/journal.pone.0345117)
Supplement: S5 Data — (ZIP) [file pone.0345117.s005.zip › S6_ Code (state utils)/cal_lr.docx]

# Copyright 2025 imoneoi and the LlamaFactory team.

#

# This code is inspired by the imoneoi's OpenChat library.

# https://github.com/imoneoi/openchat/blob/3.6.0/ochat/training_deepspeed/train.py

#

# Licensed under the Apache License, Version 2.0 (the "License");

# you may not use this file except in compliance with the License.

# You may obtain a copy of the License at

#

# http://www.apache.org/licenses/LICENSE-2.0

#

# Unless required by applicable law or agreed to in writing, software

# distributed under the License is distributed on an "AS IS" BASIS,

# WITHOUT WARRANTIES OR CONDITIONS OF ANY KIND, either express or implied.

# See the License for the specific language governing permissions and

# limitations under the License.

import math

from typing import Literal

import fire

import torch

from torch.utils.data import DataLoader

from tqdm import tqdm

from transformers import DataCollatorForLanguageModeling

from llamafactory.data import MultiModalDataCollatorForSeq2Seq, get_dataset, get_template_and_fix_tokenizer

from llamafactory.extras.constants import IGNORE_INDEX

from llamafactory.hparams import get_train_args

from llamafactory.model import load_tokenizer

BASE_LR = 3e-4 # 1.5e-4 for 30B-70B models

BASE_BS = 4_000_000 # from llama paper

def calculate_lr(

model_name_or_path: str,

batch_size: int, # total batch size, namely (batch size * gradient accumulation * world size)

stage: Literal["pt", "sft"] = "sft",

dataset: str = "alpaca_en_demo",

dataset_dir: str = "data",

template: str = "default",

cutoff_len: int = 2048, # i.e. maximum input length during training

is_mistral_or_gemma: bool = False, # mistral and gemma models opt for a smaller learning rate,

packing: bool = False,

):

r"""Calculate the optimal learning rate for 7B/13B models using LLaMA's hyper-parameters.

Usage:

python cal_lr.py --model_name_or_path path_to_model --dataset alpaca_en_demo --cutoff_len 1024 --batch_size 16

"""

model_args, data_args, training_args, _, _ = get_train_args(

dict(

stage=stage,

model_name_or_path=model_name_or_path,

dataset=dataset,

dataset_dir=dataset_dir,

template=template,

cutoff_len=cutoff_len,

packing=packing,

preprocessing_num_workers=16,

output_dir="dummy_dir",

overwrite_cache=True,

do_train=True,

)

)

tokenizer_module = load_tokenizer(model_args)

tokenizer = tokenizer_module["tokenizer"]

template = get_template_and_fix_tokenizer(tokenizer, data_args)

trainset = get_dataset(template, model_args, data_args, training_args, stage, **tokenizer_module)["train_dataset"]

if stage == "pt":

data_collator = DataCollatorForLanguageModeling(tokenizer=tokenizer, mlm=False)

elif stage == "sft":

data_collator = MultiModalDataCollatorForSeq2Seq(

template=template, tokenizer=tokenizer, label_pad_token_id=IGNORE_INDEX

)

else:

raise NotImplementedError(f"Stage does not supported: {stage}.")

dataloader = DataLoader(trainset, batch_size, shuffle=False, collate_fn=data_collator, pin_memory=True)

valid_tokens, total_tokens = 0, 0

for batch in tqdm(dataloader, desc="Collecting valid tokens"):

valid_tokens += torch.sum(batch["labels"] != IGNORE_INDEX).item()

total_tokens += torch.numel(batch["labels"])

valid_ratio = valid_tokens / total_tokens

token_batch_size = cutoff_len * batch_size * valid_ratio

lr = BASE_LR * math.sqrt(token_batch_size / BASE_BS) # lr ~ sqrt(batch_size)

lr = lr / 6.0 if is_mistral_or_gemma else lr

print(

f"Optimal learning rate is {lr:.2e} for valid ratio% {valid_ratio * 100:.2f} "

f"and effective token batch size {token_batch_size:.2f}"

)

if __name__ == "__main__":

fire.Fire(calculate_lr)
