# Supplementary material for: Research on the development of an automated system for psychology questionnaire generation based on large language models
Source: PLoS One. 2026 Apr 24;21(4):e0345117. doi: 10.1371/journal.pone.0345117 (PMC13108753; doi:10.1371/journal.pone.0345117)
Supplement: S5 Data — (ZIP) [file pone.0345117.s005.zip › S6_ Code (state utils)/cal_mfu.docx]

# Copyright 2025 the LlamaFactory team.

#

# Licensed under the Apache License, Version 2.0 (the "License");

# you may not use this file except in compliance with the License.

# You may obtain a copy of the License at

#

# http://www.apache.org/licenses/LICENSE-2.0

#

# Unless required by applicable law or agreed to in writing, software

# distributed under the License is distributed on an "AS IS" BASIS,

# WITHOUT WARRANTIES OR CONDITIONS OF ANY KIND, either express or implied.

# See the License for the specific language governing permissions and

# limitations under the License.

import json

import os

import fire

import torch

import torch.distributed as dist

from transformers import AutoConfig

from llamafactory.train.tuner import run_exp

BASE = 2 # gemm (add + mul)

def compute_model_flops(

model_name_or_path: str,

total_batch_size: int,

seq_length: int,

include_backward: bool = True,

include_recompute: bool = False,

include_flashattn: bool = False,

) -> int:

r"""Calculate the FLOPs of model per forward/backward pass."""

config = AutoConfig.from_pretrained(model_name_or_path)

hidden_size = getattr(config, "hidden_size", None)

vocab_size = getattr(config, "vocab_size", None)

intermediate_size = getattr(config, "intermediate_size", None)

num_attention_heads = getattr(config, "num_attention_heads", None)

num_key_value_heads = getattr(config, "num_key_value_heads", None)

num_hidden_layers = getattr(config, "num_hidden_layers", None)

tie_word_embeddings = getattr(config, "tie_word_embeddings", False)

# mlp module

mlp_flops_per_token = 3 * BASE * hidden_size * intermediate_size # up, gate, down

mlp_flops = total_batch_size * seq_length * num_hidden_layers * mlp_flops_per_token

# attn projector module

q_flops_per_token = BASE * hidden_size * hidden_size

o_flops_per_token = BASE * hidden_size * hidden_size

k_flops_per_token = BASE * hidden_size * hidden_size * num_key_value_heads // num_attention_heads

v_flops_per_token = BASE * hidden_size * hidden_size * num_key_value_heads // num_attention_heads

attn_proj_flops_per_token = q_flops_per_token + o_flops_per_token + k_flops_per_token + v_flops_per_token

attn_proj_flops = total_batch_size * seq_length * num_hidden_layers * attn_proj_flops_per_token

# attn sdpa module

sdpa_flops_per_layer = 2 * BASE * hidden_size * seq_length * seq_length # (q * k^T) * v

sdpa_flops = total_batch_size * num_hidden_layers * sdpa_flops_per_layer

# embedding module

embedding_flops_per_token = hidden_size * vocab_size

embedding_flops = total_batch_size * seq_length * embedding_flops_per_token

if tie_word_embeddings is False:

embedding_flops *= 2

non_embedding_flops = mlp_flops + attn_proj_flops + sdpa_flops

non_embedding_coeff, embedding_coeff = 1, 1

if include_backward:

non_embedding_coeff += 2

embedding_coeff += 2

if include_recompute:

non_embedding_coeff += 1

total_flops = non_embedding_coeff * non_embedding_flops + embedding_coeff * embedding_flops

if include_flashattn:

total_flops += sdpa_flops

return total_flops

def compute_device_flops(world_size: int) -> float:

r"""Calculate the FLOPs of the device capability per second."""

device_name = torch.cuda.get_device_name()

if "H100" in device_name or "H800" in device_name:

return 989 * 1e12 * world_size

elif "A100" in device_name or "A800" in device_name:

return 312 * 1e12 * world_size

elif "V100" in device_name:

return 125 * 1e12 * world_size

elif "4090" in device_name:

return 98 * 1e12 * world_size

else:

raise NotImplementedError(f"Device not supported: {device_name}.")

def calculate_mfu(

model_name_or_path: str,

batch_size: int = 1,

seq_length: int = 1024,

num_steps: int = 100,

finetuning_type: str = "lora",

flash_attn: str = "auto",

deepspeed_stage: int = 0,

disable_gc: bool = False,

liger_kernel: bool = False,

unsloth_gc: bool = False,

) -> float:

r"""Calculate MFU for given model and hyper-params.

Usage: python cal_mfu.py --model_name_or_path path_to_model --batch_size 1 --seq_length 1024

"""

args = {

"model_name_or_path": model_name_or_path,

"flash_attn": flash_attn,

"disable_gradient_checkpointing": disable_gc,

"enable_liger_kernel": liger_kernel,

"use_unsloth_gc": unsloth_gc,

"stage": "pt",

"do_train": True,

"finetuning_type": finetuning_type,

"dataset": "c4_demo",

"cutoff_len": seq_length,

"output_dir": os.path.join("saves", "test_mfu"),

"logging_strategy": "no",

"save_strategy": "no",

"save_only_model": True,

"overwrite_output_dir": True,

"per_device_train_batch_size": batch_size,

"max_steps": num_steps,

"bf16": True,

}

if deepspeed_stage in [2, 3]:

args["deepspeed"] = f"examples/deepspeed/ds_z{deepspeed_stage}_config.json"

run_exp(args)

if dist.is_initialized():

dist.barrier()

world_size = dist.get_world_size()

else:

world_size = 1

if int(os.getenv("LOCAL_RANK", "0")) == 0:

with open(os.path.join("saves", "test_mfu", "all_results.json"), encoding="utf-8") as f:

result = json.load(f)

total_batch_size = batch_size * world_size

mfu_value = (

result["train_steps_per_second"]

* compute_model_flops(model_name_or_path, total_batch_size, seq_length)

/ compute_device_flops(world_size)

)

print(f"MFU: {mfu_value * 100:.2f}%")

if __name__ == "__main__":

fire.Fire(calculate_mfu)
