# Supplementary material for: Research on the development of an automated system for psychology questionnaire generation based on large language models
Source: PLoS One. 2026 Apr 24;21(4):e0345117. doi: 10.1371/journal.pone.0345117 (PMC13108753; doi:10.1371/journal.pone.0345117)
Supplement: S5 Data — (ZIP) [file pone.0345117.s005.zip › S6_ Code (state utils)/cal_ppl.docx]

# Copyright 2025 the LlamaFactory team.

#

# Licensed under the Apache License, Version 2.0 (the "License");

# you may not use this file except in compliance with the License.

# You may obtain a copy of the License at

#

# http://www.apache.org/licenses/LICENSE-2.0

#

# Unless required by applicable law or agreed to in writing, software

# distributed under the License is distributed on an "AS IS" BASIS,

# WITHOUT WARRANTIES OR CONDITIONS OF ANY KIND, either express or implied.

# See the License for the specific language governing permissions and

# limitations under the License.

import json

from dataclasses import dataclass

from typing import Any, Literal

import fire

import torch

from torch.utils.data import DataLoader

from tqdm import tqdm

from transformers import DataCollatorForLanguageModeling

from llamafactory.data import MultiModalDataCollatorForSeq2Seq, get_dataset, get_template_and_fix_tokenizer

from llamafactory.extras.constants import IGNORE_INDEX

from llamafactory.hparams import get_train_args

from llamafactory.model import load_model, load_tokenizer

@dataclass

class PairwiseDataCollatorWithPadding(MultiModalDataCollatorForSeq2Seq):

r"""Data collator for pairwise data."""

train_on_prompt: bool = False

def __call__(self, features: list[dict[str, Any]]) -> dict[str, torch.Tensor]:

r"""Pad batched data to the longest sequence in the batch."""

chosen_features = []

for feature in features:

chosen_features.append(

{

"input_ids": feature["chosen_input_ids"],

"attention_mask": feature["chosen_attention_mask"],

"labels": feature["chosen_input_ids"] if self.train_on_prompt else feature["chosen_labels"],

"images": feature["images"],

"videos": feature["videos"],

"audios": feature["audios"],

}

)

return super().__call__(chosen_features)

def calculate_ppl(

model_name_or_path: str,

save_name: str = "ppl.json",

batch_size: int = 4,

stage: Literal["pt", "sft", "rm"] = "sft",

dataset: str = "alpaca_en_demo",

dataset_dir: str = "data",

template: str = "default",

cutoff_len: int = 2048,

max_samples: int | None = None,

train_on_prompt: bool = False,

):

r"""Calculate the ppl on the dataset of the pre-trained models.

Usage: export CUDA_VISIBLE_DEVICES=0

python cal_ppl.py --model_name_or_path path_to_model --dataset alpaca_en_demo --save_name ppl.json

"""

model_args, data_args, training_args, finetuning_args, _ = get_train_args(

dict(

stage=stage,

model_name_or_path=model_name_or_path,

dataset=dataset,

dataset_dir=dataset_dir,

template=template,

cutoff_len=cutoff_len,

max_samples=max_samples,

train_on_prompt=train_on_prompt,

preprocessing_num_workers=16,

output_dir="dummy_dir",

overwrite_cache=True,

do_train=True,

)

)

tokenizer_module = load_tokenizer(model_args)

tokenizer = tokenizer_module["tokenizer"]

template = get_template_and_fix_tokenizer(tokenizer, data_args)

trainset = get_dataset(template, model_args, data_args, training_args, stage, **tokenizer_module)["train_dataset"]

model = load_model(tokenizer, model_args, finetuning_args, is_trainable=False)

if stage == "pt":

data_collator = DataCollatorForLanguageModeling(tokenizer=tokenizer, mlm=False)

elif stage == "sft":

data_collator = MultiModalDataCollatorForSeq2Seq(

template=template, tokenizer=tokenizer, label_pad_token_id=IGNORE_INDEX

)

elif stage == "rm":

data_collator = PairwiseDataCollatorWithPadding(

template=template, tokenizer=tokenizer, label_pad_token_id=IGNORE_INDEX, train_on_prompt=train_on_prompt

)

else:

raise NotImplementedError(f"Stage does not supported: {stage}.")

dataloader = DataLoader(trainset, batch_size, shuffle=False, collate_fn=data_collator, pin_memory=True)

criterion = torch.nn.CrossEntropyLoss(reduction="none")

total_ppl = 0

perplexities = []

batch: dict[str, torch.Tensor]

with torch.no_grad():

for batch in tqdm(dataloader, desc="Computing perplexities"):

batch = batch.to(model.device)

outputs = model(**batch)

shift_logits: torch.Tensor = outputs["logits"][..., :-1, :]

shift_labels: torch.Tensor = batch["labels"][..., 1:]

loss_mask = shift_labels != IGNORE_INDEX

flatten_logits = shift_logits.contiguous().view(shift_labels.size(0) * shift_labels.size(1), -1)

flatten_labels = shift_labels.contiguous().view(-1)

token_logps: torch.Tensor = criterion(flatten_logits, flatten_labels)

token_logps = token_logps.contiguous().view(shift_logits.size(0), -1)

sentence_logps = (token_logps * loss_mask).sum(-1) / loss_mask.sum(-1)

total_ppl += sentence_logps.exp().sum().item()

perplexities.extend(sentence_logps.exp().tolist())

with open(save_name, "w", encoding="utf-8") as f:

json.dump(perplexities, f, indent=2)

print(f"Average perplexity is {total_ppl / len(perplexities):.2f}")

print(f"Perplexities have been saved at {save_name}.")

if __name__ == "__main__":

fire.Fire(calculate_ppl)
