# Supplementary material for: Research on the development of an automated system for psychology questionnaire generation based on large language models
Source: PLoS One. 2026 Apr 24;21(4):e0345117. doi: 10.1371/journal.pone.0345117 (PMC13108753; doi:10.1371/journal.pone.0345117)
Supplement: S5 Data — (ZIP) [file pone.0345117.s005.zip › S6_ Code (state utils)/length_cdf.docx]

# Copyright 2025 the LlamaFactory team.

#

# Licensed under the Apache License, Version 2.0 (the "License");

# you may not use this file except in compliance with the License.

# You may obtain a copy of the License at

#

# http://www.apache.org/licenses/LICENSE-2.0

#

# Unless required by applicable law or agreed to in writing, software

# distributed under the License is distributed on an "AS IS" BASIS,

# WITHOUT WARRANTIES OR CONDITIONS OF ANY KIND, either express or implied.

# See the License for the specific language governing permissions and

# limitations under the License.

from collections import defaultdict

import fire

from tqdm import tqdm

from llamafactory.data import get_dataset, get_template_and_fix_tokenizer

from llamafactory.hparams import get_train_args

from llamafactory.model import load_tokenizer

def length_cdf(

model_name_or_path: str,

dataset: str = "alpaca_en_demo",

dataset_dir: str = "data",

template: str = "default",

interval: int = 1000,

):

r"""Calculate the distribution of the input lengths in the dataset.

Usage: export CUDA_VISIBLE_DEVICES=0

python length_cdf.py --model_name_or_path path_to_model --dataset alpaca_en_demo --template default

"""

model_args, data_args, training_args, _, _ = get_train_args(

dict(

stage="sft",

model_name_or_path=model_name_or_path,

dataset=dataset,

dataset_dir=dataset_dir,

template=template,

cutoff_len=1_000_000,

preprocessing_num_workers=16,

output_dir="dummy_dir",

overwrite_cache=True,

do_train=True,

)

)

tokenizer_module = load_tokenizer(model_args)

template = get_template_and_fix_tokenizer(tokenizer_module["tokenizer"], data_args)

trainset = get_dataset(template, model_args, data_args, training_args, "sft", **tokenizer_module)["train_dataset"]

total_num = len(trainset)

length_dict = defaultdict(int)

for sample in tqdm(trainset["input_ids"], desc="Collecting lengths"):

length_dict[len(sample) // interval * interval] += 1

length_tuples = list(length_dict.items())

length_tuples.sort()

count_accu, prob_accu = 0, 0

for length, count in length_tuples:

count_accu += count

prob_accu += count / total_num * 100

print(f"{count_accu:d} ({prob_accu:.2f}%) samples have length < {length + interval}.")

if __name__ == "__main__":

fire.Fire(length_cdf)
